# Temporal dependence emerges from a carbodiimide driven acid–anhydride equilibrium coupled to a photocatalytic decarboxylation reaction

**DOI:** 10.1039/d6sc03290g

**Published:** 2026-07-07

**Authors:** Rens Ham, Bettina Baumgartner, Joost N. H. Reek

**Affiliations:** a Van 't Hoff Institute for Molecular Sciences, University of Amsterdam 1098 XH Amsterdam The Netherlands j.n.h.reek@uva.nl

## Abstract

Neurons process multiple stimuli in a temporally dependent manner, which filters noise and enables precise control over complex functions. Synthetically mimicking neuronal processes may be useful for autonomous chemical systems that are able to make their own decisions, yet such time-dependence has not found an equivalent in synthetic reaction networks. Herein, we show that carbodiimide driven equilibria between acid and anhydride can be coupled to photochemical decarboxylation from which temporal input dependence emerges. Under optimized conditions, the decarboxylation of the anhydride is much faster than the acid. However, the anhydride hydrolyses back to the acid, and as such is only temporally present after carbodiimide addition, which limits its conversion to this time period. The network therefore displays temporal dependence, as the product is only generated when both carbodiimide and light inputs align in time. Such systems provide a platform to explore the role of temporal gating in biological regulation and offer new strategies for adaptive catalysis and chemical computing.

## Abstract 2

Temporal input dependence emerges from a chemical reaction network containing a carbodiimide driven equilibrium coupled to a photochemical decarboxylation.

## Graphical abstract

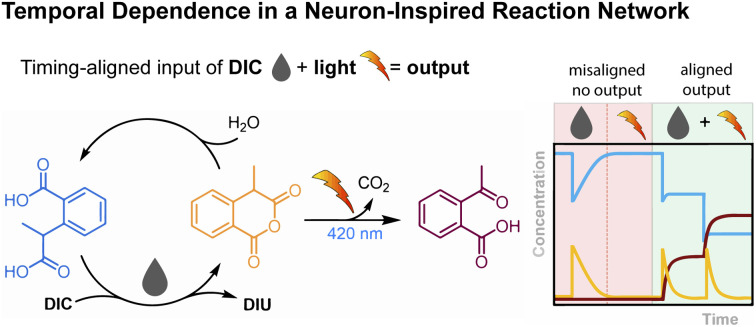

## Introduction

**Fig. 1:**
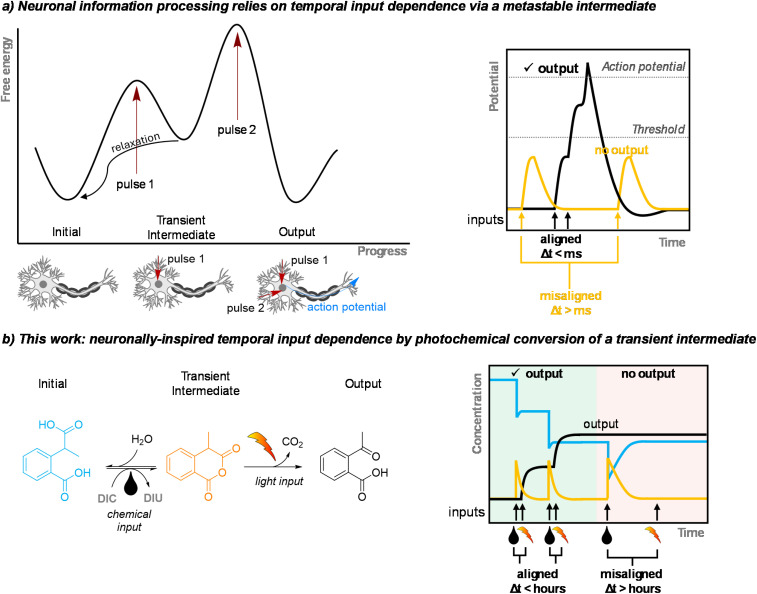
Schematic representation of (a) the neuronal membrane potential upon input of electrical pulses with a schematic of the reaction coordinate and the membrane potential over time when agitated with aligned (black) and misaligned (orange) input, and (b) the chemical reaction network inspired by neuronal processing that exhibits temporal input dependence presented in this work and the corresponding schematic concentration over time plot with initial acid (in blue), intermediate anhydride (in orange), and output ketone (in black) upon aligned (left side, in green) and misaligned (right side, in red) input of carbodiimide and light. Colours of the lines correspond to the concentration of the chemical compounds in the same colour.

The development of synthetic chemical systems that can make their own decisions would revolutionise current chemical practice. This requires synthetic systems that are capable of rudimentary brain-functions and information processing from multiple signals. Natural information processing occurs in the neurons that process electric signals only when multiple inputs arrive within a limited timeframe by generating an action potential, which is important for learning and memory functions.^1^ The processing phenomenon that occurs in neurons is temporal coincidence, which covers temporal summation and coincidence detection.^2^ Temporal summation (Fig. 1a) is the integration of input over time, where the first input generates a metastable, short-lived (about 15 ms) intermediate state which dissipates back to the initial state unless rapidly followed by the second input in which case the threshold for a signal is reached.^3^ Coincidence detection has a programmed time window which the system considers as simultaneous, and when sufficient signals arrive within this window, the threshold is reached without intermediate state(s). Due to the fast timescales of temporal summation, it approaches true coincidence detection and as such their differentiation is difficult to determine.^4^ Crucially, in both cases the signals are processed in a time-dependent manner, which for neural functions is important because it filters noise and provides high fidelity over signalling. Developing synthetic systems that display time-dependent features are thus relevant for chemical computing, and adaptive and autonomous (catalytic) systems containing lifelike features.^5–7^ Therefore we sought to develop a synthetic system that is conceptually similar to temporal coincidence in neuronal processing, yet at different timescales to make it relevant for synthetic applications.

In essence, neuronal information processing resembles an AND-gate found in molecular logic gates,^8–10^ combined with a temporal alignment requirement that emerges from the input-gated conversion of a metastable intermediate. The lifetime of this intermediate determines the processing speed, which must be fast in neurons for the body to respond quickly. However, this is not the case for synthetic applications as timescales for information processing in molecular synthesis are different. As such, it is valuable to have control over the speed at which processing occurs so that it can match time windows of chemical conversion. For these synthetic applications it is therefore crucial to have control over the lifetime of the metastable intermediate.

Introducing reversible control in synthetic chemical reaction networks can be achieved by inputs that switch an equilibrium to a different state, such as light, pH, redox, and chemical inputs. “Fuelled equilibria” (Fig. 1b) consist of a dynamic equilibrium that can be temporally shifted to a different chemical state by a chemical input (coined the fuel) with high control.^11–14^ Crucially, such systems generate a metastable intermediate that relaxes back to the initial state if not agitated again. Specifically, the carbodiimide-driven equilibrium between acid and anhydride (Fig. 1b), developed by the groups of Hartley and Boekhoven,^15,16^ has been successful with a typical read-out associated to a self-assembly or polymerisation process,^17–22^ and even to facilitate an endergonic Diels–Alder reaction.^23^ We recently reported the coupling of the acid–anhydride equilibrium to an orthogonal catalytic reaction, by using the acid and anhydride as different allosteric effectors that modulate catalytic activity of Pt_2_L_4_ cages by binding these components.^24^

**Fig. 2:**
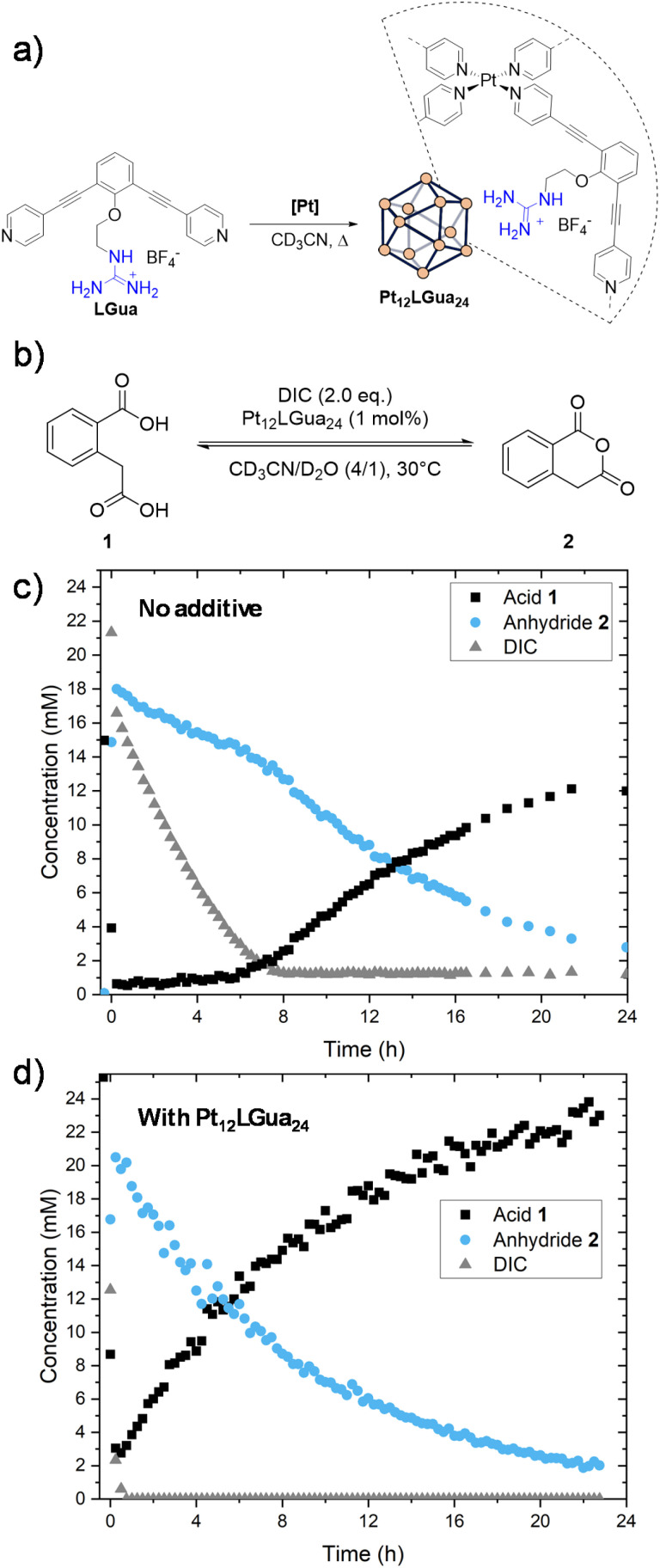
(a) Reaction scheme for preparation of Pt_12_LGua_24_*via* self-assembly. (b) Reaction scheme for equilibrium between homophthalic acid (1) and homophthalic anhydride (2), “fuelled” by DIC. (c) Reaction profile for the DIC-driven equilibrium between 1 and 2 in absence of nanosphere. (d) Reaction profile for the DIC-driven equilibrium between 1 and 2 in presence of Pt_12_LGua_24_ (SI Section 2).

The examples above have in common that the carbodiimide addition is used as the sole input to control the network, after which it equilibrates autonomously without further external control.^25^ Although the combination of other inputs has recently proven fruitful,^26–31^ we were particularly interested to explore multiple stimuli by combining the carbodiimide driven equilibrium with light as an additional input as it can easily be tuned and provides external spatiotemporal control.^32,33^ Such advantages have recently been recognised and utilised in photoinitiated networks based on transient self-assembly,^34^ and enzymatic networks.^35^ The groups of Hartley and Konkolewicz reported AND-gated transient crosslinking of polymers,^36^ in which light was used to initiate the network, therefore not showing temporal dependence on the inputs. Instead, when the tuneable dynamic equilibrium is coupled to a non-reversible photocatalytic step it should provide temporal dependence because this generates a metastable intermediate that can be irreversibly converted further by an orthogonal stimulus (Fig. 1b). Specifically photochemical decarboxylation reactions have been well developed,^37–39^ and we recently reported the photochemical decarboxylative oxygenation reaction using Pt_12_LGua_24_ nanospheres (Fig. 2a) as photocatalyst.^40^

Herein, we report a system that shows temporal input dependence by coupling the *N*,*N*-diisopropylcarbodiimide (DIC) driven acid–anhydride equilibrium with a photocatalytic decarboxylation reaction (Fig. 1b). In a mixture of CH_3_CN/H_2_O (4/1), Pt_12_LGua_24_ acts as photocatalyst for the decarboxylation of the anhydride, which is substantially faster than the decarboxylation of acid substrate and the anhydride hydrolysis reaction to reform the diacid. When these reactions are coupled together in the same system, they constitute a chemical reaction network that displays temporal dependence, only providing the photochemical product when both DIC and light are provided within the temporal overlap window.

## Results and discussion

To obtain temporal dependence, two separately controlled processes – the acid–anhydride equilibrium and photodecarboxylation – have to be coupled, which in this work were first investigated and optimised separately.

### Acid–anhydride equilibrium

We started investigating homophthalic acid (1) as model substrate (Fig. 2b), as it could undergo both anhydride formation to homophthalic anhydride (2) and the decarboxylative oxygenation to form the carbonyl product. The Boekhoven group showed that diacids undergo reversible anhydride formation, without losing much material as *N*-acylurea side product.^41^ Typically, 2-(*N*-morpholino)ethanesulfonic acid (MES) is added as buffering agent in high concentrations, however, MES is incompatible with oxidative photochemistry as the amines can quench photoexcited states (Table S6). Therefore, we explored conditions for this equilibrium in absence of buffer (Fig. 2b). When typical conditions are used in which 2 equivalents of DIC with respect to diacid 1 are added in CD_3_CN/D_2_O (4/1) at 30 °C,^24^ quantitative conversion to anhydride 2 occurs within 15 minutes (Fig. 2c). In the first 7 hours hardly any acid reforms by hydrolysis, whereas the anhydride concentration already declines (Fig. 2c). When DIC is fully consumed (after 7 hours), the formation of the diacid and decline of the anhydride progress at approximately the same speed. At the end of the experiment only 61% of 1 is retained (black boxes), indicating that under these conditions the equilibrium is not fully reversible due to side product formation.^41^

Interestingly, when the same experiment is performed in the presence of 1 mol% (*versus*1) of Pt_12_LGua_24_, the equilibrium reaction is reversible and 1 is fully recovered at the end of the reaction (Fig. 2d). Importantly, when Pt_12_LGua_24_ is present, the acid reforms almost instantly (Fig. 2d, black boxes), whereas 1 is not observable for 7 hours in absence of nanosphere (Fig. 2c, black boxes). The rate of anhydride hydrolysis is unaffected by the nanosphere (Table S1). However, control experiments show that Pt_12_LGua_24_ catalyses the hydrolysis of excess DIC to *N*,*N*-diisopropylurea (DIU), converting the unreacted DIC at the start of the reaction, allowing immediate acid reformation. Monitoring the DIC to DIU conversion during the experiment shows that DIC is consumed an order of magnitude faster in the presence of the nanosphere (Table S1). Also, a Pt_12_L_24_ nanosphere that does not contain guanidinium groups and the free guanidinium salt (Table S1) accelerated the DIC to DIU conversion rate compared to the non-catalysed system, but not as much as Pt_12_LGua_24_. We believe that the basicity of DIC allows H-bonding with the guanidinium groups to catalyse its hydrolysis, whereas anhydride 2 is much less basic and therefore its hydrolysis is unaffected. Importantly, in the current experiments, the DIC reacts rapidly with the diacid to form the anhydride and the excess DIC is reacted away quickly by the Pt_12_LGua_24_ nanosphere, leading to less side product formation.

**Fig. 3:**
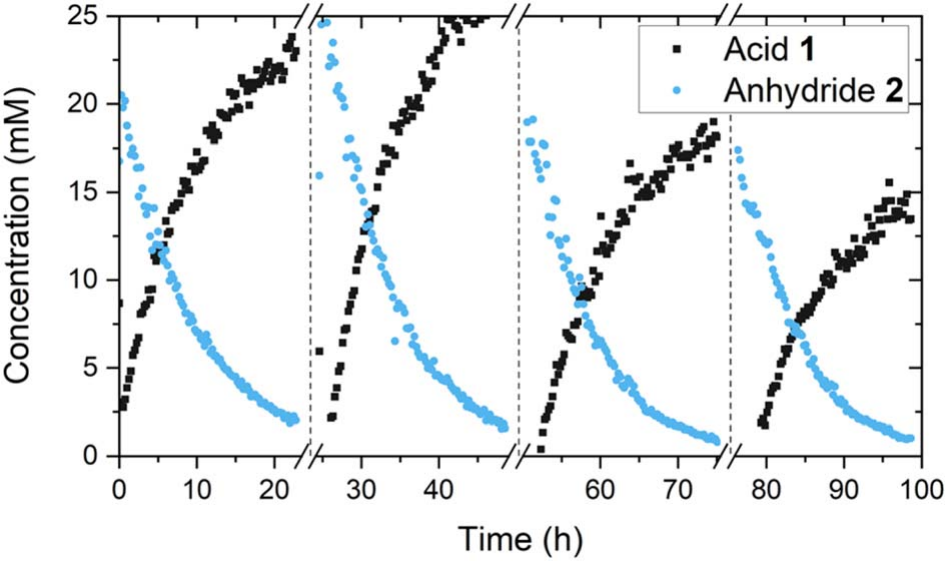
Reaction profiles for the equilibrium between 1 and 2 in presence of Pt_12_LGua_24_ (1 mol%), breaks indicate addition of DIC (2.0 eq.).

To show the reversibility of the diacid anhydride cycle, DIC was added multiple times and the reaction was monitored over time (Fig. 3). The breaks indicate consecutive DIC additions (2.0 eq. for each addition). The reaction profile is repeated four times, with minor fatigue showing, *i.e.* the total acid–anhydride concentration reduces to some extend (about 20%) after the third and fourth addition due to formation of *N*-acylurea,^41^ which continues to increase upon more additions of DIC. In contrast, when the nanosphere was not present, no acid nor anhydride is left after four cycles (Fig. S13). The selectivity for one cycle was determined from Fig. 2c and d by determining the amount of recovered acid 1 after 24 hours,^41^ which shows a selectivity of 61% and 90%, without and with Pt_12_LGua_24_, respectively. Additionally, acid 3 was also investigated under the same conditions, which was found to have an improved selectivity of 96% (Fig. S14–16). Thus, the nanosphere significantly improves the selectivity of the acid–anhydride equilibrium, allowing it to go through multiple cycles.

### Photocatalytic decarboxylation

Having established that the acid–anhydride concentrations can reversibly be forced out-of-equilibrium by DIC, we required a second input that could be incorporated within the same system. Recently, we reported the photochemical decarboxylation to generate benzylic radicals *via* reductive quenching of Pt_12_LGua_24_.^40^ Exposing diacid 1 to these conditions resulted in a mixture of oxidation products. As such, we selected 3 as model substrate for the photochemical reactions and exposed it to Pt_12_LGua_24_ (1 mol%) in MeCN and 390 nm LED irradiation, which yielded 78% of the expected 2-acetylbenzoic acid (4) (Table 1, entry 1). When anhydride 5 was exposed to the same reaction conditions, 4 was also obtained in 70% (entry 2). These conditions therefore do not result in specific conversion of either substrate, and as such, the addition of DIC will have little influence on the reaction outcome, *i.e.* the reaction only responds to light as input. We hypothesised that the substrate specificity can be improved by reducing the reductive quenching efficiency of acid 3 by reducing its affinity for the guanidine binding site, which is possible by addition of H_2_O (Fig. S21 and S22). Indeed, in CH_3_CN/H_2_O (4/1), the photocatalytic conversion of acid 3 to product 4 is slow (entry 3), whereas the conversion of the anhydride 5 is fast, yielding 86% of 4 (entry 4). Crucially, this shows that the reaction is substrate specific in CH_3_CN/H_2_O (4/1), meaning that DIC can be used as input to promote formation of anhydride 5, which in turn modulates the photochemical activity.

**Table 1:** Photocatalytic decarboxylation of methyl-homophthalic acid (3) or methyl-homophthalic anhydride (5) to form 2-acetylbenzoic acid (4) using Pt_12_LGua_24_ as photocatalyst^a^

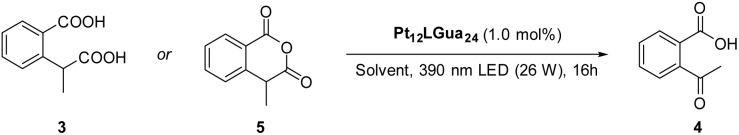			
Entry	Substrate	CH_3_CN/H_2_O	Yield 4 (%)^b^
1	Acid 3	1/0	78
2	Anhydride 5	1/0	70
3	Acid 3	4/1	**8**
4	Anhydride 5	4/1	**86**
5	3 or 5	1/0 or 4/1	n.d.
6^d^	3 or 5	1/0 or 4/1	n.d.

a Conditions: diacid or anhydride (2.5 µmol, 1.0 eq.) and Pt_12_LGua_24_ (1.0 mol%) in a ratio of CH_3_CN/H_2_O (0.5 mL) were irradiated with a 390 nm Kessil lamp (26 W, 50%) for 16 h (SI Section 3).

b Determined by GC/MS-FID integration against 1,3,5-trimethoxybenzene as external standard after calibration.

c In the dark at 40 °C.

d Without Pt_12_LGua_24_. n.d. = not detected.

Control experiments showed that the reaction does not proceed without Pt_12_LGua_24_ or light (Table 1, entries 5 and 6). As previously reported, in CH_3_CN acid 3 reductively quenches the excited state to form carbon radicals that react with O_2_ to form 4,^40^ a process that is hampered in CH_3_CN/H_2_O (4/1) (Fig. S21 and S22). On the other hand, anhydride 5 does not quench the excited state of Pt_12_LGua_24_, even in CH_3_CN (Fig. S20), thus this reaction likely proceeds *via* excited state reactivity with O_2_, while still liberating CO_2_ (Fig. S27). UV-vis titrations showed that there are no significant interactions between Pt_12_LGua_24_ and either 3 or 5 (Fig. S17 and S18) in CH_3_CN/H_2_O (4/1), thus it is kinetically feasible for the excited state to react with O_2_. We previously noted that Pt_12_LGua_24_ can generate singlet oxygen (^1^O_2_),^40^ and when NaN_3_ was added to the standard conditions as ^1^O_2_ quencher, indeed no product was observed (Tables S4 and S5).^42,43^ Thus, the reductive quenching of carboxylate 3 is shut down in CH_3_CN/H_2_O (4/1), making O_2_ sensitisation dominant, which leads to a large difference in reactivity between acid 3 and anhydride 5, providing photocatalytic conditions that may be coupled to a DIC driven equilibrium.

### Temporal dependence emerges from coupling the reactions

**Fig. 4:**
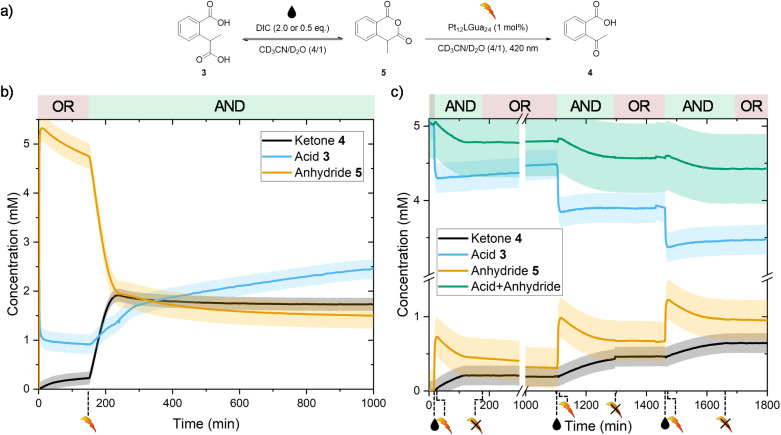
(a) Reaction scheme of the AND-gated reaction between 3, 4 and 5 with Pt_12_LGua_24_ (1 mol%) in CD_3_CN/D_2_O (4/1) under an atmosphere of O_2_ (SI Section 5). (b) Reaction profile upon addition of DIC (2.0 eq.) at *t* = 1 min and light irradiation at the dotted line. (c) Reaction profile of coupled process between 3, 4 and 5 with Pt_12_LGua_24_ (1 mol%) upon addition of DIC (0.5 eq.) as indicated by the black droplets and light irradiation as shown by the pictograms. The cross indicates that the light was switched off. The error margin is shown as shaded area.

With suitable conditions in hand, we explored the coupling of the photochemical reaction with the acid–anhydride equilibrium. The reaction progress was monitored by Fourier-transfrom infrared (FT-IR) spectroscopy, taking advantage of the distinct carbonyl stretching bands of acid 3, ketone 4, and anhydride 5, which enabled simultaneous quantification of all species by partial least squares (PLS) regression (SI Section 5).^44^ Briefly, a solution of 3 (5 mM) in CD_3_CN/D_2_O (4/1) with Pt_12_LGua_24_ (1 mol%) under an atmosphere of O_2_ was stirred in front of a 420 nm light source while being continuously circulated through a liquid IR transmission cell (Fig. 4a and S30). To switch from 3 to 5, DIC (2.0 eq.) was added (at *t* = 0 minutes) to the solution to quickly form 5 (Fig. 4b), and the hydrolysis was monitored in the dark for 150 minutes. During this period, the system is in OR configuration as the solution is not irradiated, and as such ketone 4 is not produced. To go into AND configuration, the 420 nm light source was switched on (dotted line, Fig. 4b). Instantly, the system reacts to this perturbation (indicated by the kink), and 4 starts to rapidly form. Under these conditions, the formation of 4 comes to a halt at 35% yield, which was also confirmed by GC-MS after the reaction (Fig. S36–39). When only light is provided, the ketone product does not significantly form (Fig. S38). This shows that the system works as logic AND-gate for a single perturbation.

We then aimed to control the rate of ketone formation for several times by switching from OR to the AND input states. The system was first agitated with light irradiation for 10 minutes, which did not lead to any product formation because there is no anhydride present (Fig. 4c). Then, the reaction was agitated with small amounts of DIC (0.5 eq., black droplets, Fig. 4c) to spike anhydride concentrations for short amounts of time. Since DIC decomposes upon direct irradiation, the light was off for 5 minutes after each addition so that all DIC was consumed, after which the mixture was irradiated for 180 minutes. After DIC addition, the photochemical reaction accelerates significantly and rapid consumption of anhydride 5 (Fig. 4c) occurs. At the same time, ketone 4 forms, whereas the acid 3 hardly increases, showing that under these conditions, the kinetics of anhydride hydrolysis do not compete with the photochemical conversion. At approximately 180 minutes the system relaxes to the OR state, leading to a stable equilibrium until the second injection of DIC (at around 1100 minutes). Upon injection, the profile was repeated and the system responds in a similar fashion (Fig. 4c), *i.e.* instant conversion of the acid to the anhydride, leading to the AND state and slowly converting the anhydride to the ketone over the course of 180 minutes, after which the system is in the OR state again. Thus, the photocatalytic rate is controlled by the presence of anhydride and as such the addition of DIC, providing an AND-gated process with two controllable handles that must be aligned for product formation.

After carbodiimide input, there is a maximum time within which photochemical conversion can take place, *i.e.* before the anhydride is completely hydrolysed. This temporal overlap is highly dependent on the anhydride hydrolysis kinetics. By averaging our experiments, first-order kinetics with *k* ≈ 2.26 × 10^−3^ min^−1^ were found for anhydride 5 hydrolysis (Fig. S14–S16). With this rate constant the concentration of anhydride in time can be estimated and with that the temporal window for the photochemical follow-up reaction can be predicted by the following equation.
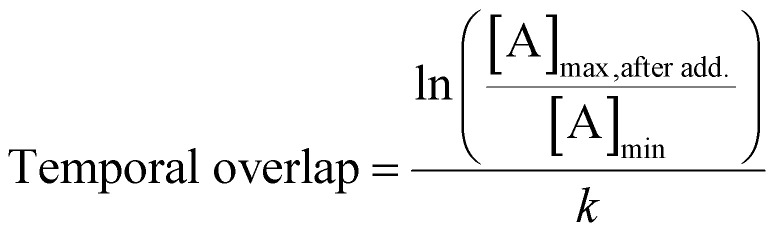


The pre-exponential factor ([A]_max,after add._) for the first-order kinetics is the maximum anhydride concentration (in mM) after DIC addition. Additionally, a minimum anhydride concentration ([A]_min_) needs to be defined below which no photochemical conversion takes place. To calculate the half-life time (*t*_1/2_), [A]_min_ is half of [A]_max,after add._, giving *t*_1/2_ = 5 hours. However, at half the concentration, photochemical conversion can typically still take place, and as such [A]_min_ can be defined as 0.1 mM. In this way, the amount of anhydride buildup is taken into account, and as such, the DIC addition in Fig. 4b ([A]_max,after add._ = 5.00 mM) has a temporal overlap of 29 hours, whereas the DIC addition in Fig. 4c ([A]_max,after add._ = 0.75 mM) has a temporal overlap of 15 hours. However, care should be taken with this formula when there is a large excess of DIC that is not efficiently hydrolysed, *e.g.* in Fig. 2c. When Pt_12_LGua_24_ is present, excess DIC is cleaned up quickly enough to have minimal disturbance. In a fundamentally similar manner, neurons have a half-life of about 5 ms,^45,46^ which the body requires to respond quickly. In synthetic systems, the desired temporal overlap is application dependent and therefore it is desirable to have a controllable temporal overlap. Since the lifetime of the metastable intermediate depends on anhydride hydrolysis kinetics, they can be designed to suit the intended application.

## Conclusion

In conclusion, we report a synthetic chemical system that displays temporal input dependence by coupling a carbodiimide driven equilibrium to a photochemical decarboxylation. The photochemical rate difference between acid 3 and anhydride 5 is large enough to only observe product 4 formation when the anhydride is present. Since the anhydride is a transient species that slowly hydrolyses to acid 3, the system is temporally dependent on two orthogonal inputs: DIC and light. The system is facilitated by Pt_12_LGua_24_, which photo-catalyses the decarboxylation and enhances the selectivity of the acid–anhydride equilibrium by hydrolysing unreacted carbodiimide. The current system is fundamentally similar to neuronal processes, although the timescales for information processing by neurons are much faster. For chemical synthesis purposes the time scales must be larger, and as the current system relies on anhydride hydrolysis, the temporal overlap can be modulated by changing the conditions into the practical time window for this application. Our work therefore provides a general route to synthetic systems that are temporally dependent on their input, and provides opportunities in chemical computing, highly controlled catalysis, and synthetic mimics of cellular signalling networks.

## Author contributions

Conceptualization: RH, JNHR; data curation: RH, BB; formal analysis: RH, BB; funding acquisition: JNHR; investigation: RH; methodology: RH, BB; project administration: JNHR; supervision: BB, JNHR; validation: RH; visualization: RH, writing – original draft: RH; writing – review and editing: RH, BB, JNHR.

## Conflicts of interest

There are no conflicts to declare.

## Supplementary Material

SC-OLF-D6SC03290G-s001

## Data Availability

Data supporting this article are included as part of the supplementary information (SI)^47–51^ and unedited data are available at figshare at https://doi.org/10.21942/uva.32041101. Supplementary information is available. See DOI: https://doi.org/10.1039/d6sc03290g.
