# Supplementary material for: Temporal dependence emerges from a carbodiimide driven acid–anhydride equilibrium coupled to a photocatalytic decarboxylation reaction
Source: Chem Sci. 2026 Jul 7. Online ahead of print. doi: 10.1039/d6sc03290g (PMC13359082; doi:10.1039/d6sc03290g)
Supplement: SC-OLF-D6SC03290G-s001 [file SC-OLF-D6SC03290G-s001.pdf]

Supplementary Information (SI) for Chemical Science.
This journal is © The Royal Society of Chemistry 2026

Temporal Dependence Emerges from a Carbodiimide Driven Acid-Anhydride Equilibrium Coupled to a Photocatalytic Decarboxylation Reaction

Rens Ham,[a] Bettina Baumgartner,[a] and Joost N.H. Reek*[a]

[a] Rens Ham MSc., dr. Bettina Baumgartner, prof. dr. Joost N.H. Reek
Van 't Hoff Institute for Molecular Sciences
University of Amsterdam
Science Park 904, 1098 XH Amsterdam
E-mail: j.n.h.reek@uva.nl

Content

1. General considerations 2
2. Equilibrium studies over time 3
3. Photocatalytic experiments 17
4. Mechanistic investigations 19
5. Online FT-IR spectroscopy 31
6. GC/MS calibration curves 42
7. Experimental references 42

1. General considerations

Reagents and solvents

All reagents were of commercial grade and used without further purification, unless noted otherwise. All reactions and procedures were performed under aerobic conditions with magnetic stirring unless noted otherwise. The synthetic preparations of **$Pt_{12}LGua_{24}$** and **$Pt_{12}LBn_{24}$** were published previously by our group, and spectral data are in accordance.[1–3] **3** and **5** were synthesized from homophthalic acid according literature procedures, spectral data are in accordance with the literature.[4,5]

NMR spectroscopy

NMR spectra were recorded on a Bruker DRX 500, Bruker AMX 400, Bruker DRX 300 or Varian Mercury 300 spectrometer at r.t. and the reported ppm values are relative to $SiMe_4$, by referencing the solvent residual peak to $SiMe_4$. Data was processed and visualized using MestReNova 15.0.1. 2D ^{1}H- Diffusion ordered spectroscopy (DOSY) spectra were recorded on a Bruker DRX 500 or Varian Mercury 300 spectrometer at 25.0°C. Before each DOSY measurement, both the temperature and the magnetic gradient were calibrated first. For ^{1}H-NMR measurements with light irradiation, a Bluepoint 4 light-source with 390-500 nm filter from Honle UV Technology was used. An optic fiber was used to irradiate the NMR sample. Data fitting and visualization was performed with MestReNova 15.0.1.

UV-Vis spectroscopy

UV-Vis spectra were recorded on a single beam Hewlett Packard 8453 spectrometer in a 1.0 cm quartz cuvette using the solvent as background at 20.0°C.

IR spectroscopy

IR spectra were recorded on a Bruker Invenio-R FTIR spectrometer with a DTGS detector using a liquid transmission cell equipped with CaF_2 windows and 200 µm PTFE spacer. The cell was connected to the reaction vessel via 1/16'' PFA tubing and supplied by a peristaltic pump. The reaction mixture was circulated through the transmission cell at a flow rate of 0.5 mL/min (see Figure S17 for scheme). Absorbance spectra were recorded with a resolution of 4 cm^{-1} and 16 scans were averaged. Spectra were calculated using a CD_3CN/D_2O (4/1) background. The spectral region between 1988 and 1670 cm^{-1} (characteristic carbonyl region) was corrected for the increasing water background by subtracting Gaussians fits to the band at 1640 cm-1 using a Matlab script. Subsequently, the spectra were transposed and further processed in Origin Pro, using partial least squares (PLS) fitting to determine the concentration of components.

Partial least squares (PLS) fitting

The PLS model was calibrated with 20 samples containing known concentrations of the components. Software used was Origin. Cross validation was applied and SVD was used as method.

GC-MS

GC-MS analysis was performed with a Shimadzu GCMS-QP2020 SE gas chromatograph mass spectrometer using the FID as detector and SH-RTX Amine column (29.0 m, 0.25 µm internal diameter, and 0.25 µm film thickness). Calibration curves were made with 1,3,5-trimethoxybenzene as reference. Typical program: injection temperature 250°C. Column oven temperature: 50°C. Ramp from 50°C to 300°C with 25°C/min, hold 300°C for 10 min.

Gas-GC

Gas product analysis was carried out with gas chromatography on an Agilent 990 Micro GC with two thermal conductivity detectors (TCD). Gas samples were analyzed over a Molsieve column (MS5A SS 10 m × 0.25 mm × 30 µm BF, Agilent) for the detection of H_2, O_2, N_2, CH_4, and CO, and over a CP188 PoraPLOT U column (Poraplot U FS 10 m × 0.25 mm × 8 µm BF, Agilent) for the detection of CO_2, ethene, ethane, propene and H_2O. For all measurements, no CH_4, ethene, ethane, or propene was detected. Operating parameters for the Molsieve column were: Ar carrier gas (Nippon gases); injection temperature 80 °C; injection time 100 ms; backflush time 15 s (CP-Porabond column Q 3 m × 0.25 mm × 3 µm, Agilent); column temperature 80 °C; static pressure mode; initial pressure 200 kPa; run time 180 seconds. Operating parameters for the PoraPLOT column were: He carrier gas (Nippon gases); injection temperature 110 °C; injection time 50 ms; backflush time 5 s (CP-Porabond column Q 3 m × 0.25 mm × 3 µm, Agilent); column temperature 70 °C; static pressure mode; initial pressure 150 kPa; run time 150 seconds. MicroGC calibration curves for H_2 and CO and CO_2 were measured using Nippon calibration gas containing H_2 (400 ppm), CO (4000 ppm), in Argon and mixing it with flow of CO_2 (Nippon). By varying the flow of calibration mixture and CO_2, several data points were collected and the calibration curve was loaded in the CDS Software.

Fluorescence spectroscopy

Fluorescence spectra were recorded on a Edinburgh Instruments FS5 spectrofluorometer using the SC-25 TE cooled sample holder to control the temperature using a xenon arc lamp for excitation. Fluoracle® was used as operating software.

2. Equilibrium studies over time

To follow the rate profiles of the acid to anhydride equilibrium, ^{1}H-NMR was used at 30°C. Briefly, a solution of homophthalic acid (10 µmol, 1.0 eq.) and 1,3,5-trimethoxybenzene (10 µmol) as internal standard in 4/1 CD_3CN/D_2O (0.5 mL, 20 mM) with additives was prepared and a spectrum was recorded at 30°C which served as t = 0. Then, DIC (3.11 µL, 20 µmol, 2.0 eq.) was added to the solution in the NMR-tube, and it was shaken thoroughly. ^{1}H-NMR spectra were recorded at set timepoints and the integrals versus the internal standard were used to determine the concentrations of the compounds over time.

Additives used and their amounts are listed in Table S1.

For multiple cycles, DIC (3.11 µL, 20 µmol, 2.0 eq.) was added after the anhydride was no longer observable.

For the determination of the rate constants, pseudo-first order kinetics are assumed since D_2O is in large excess, and as reported previously.[6] To quantify hydrolysis of **DIC**, initial rates are used as the first-order model did not always fit to the kinetic profiles.

Table S1: List of additives used for the equilibrium studies and their kinetic parameters based on the first-order kinetic model.

**Additive**	**Amount**	**$k_{hydrolysis}$ anhydride 2**	**$v_{ini, hydrolysis}$ DIC**
None	-	1.6×10^{-3} min^{-1}	5.0×10^{-2} mM/min

**$Pt_{12}LGua_{24}(BF_4)_{48}$**	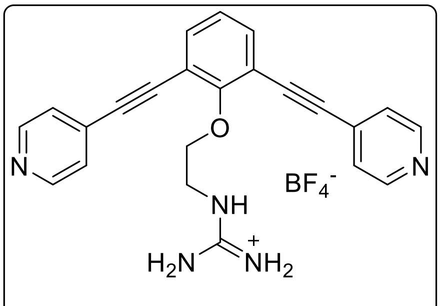 **LGua**	0.01 µmol, 1.0 mol%	1.8×10^{-3} min^{-1}	4.0×10^{-1} mM/min*
**$Pt_{12}LBn_{24}(BF_4)_{24}$**	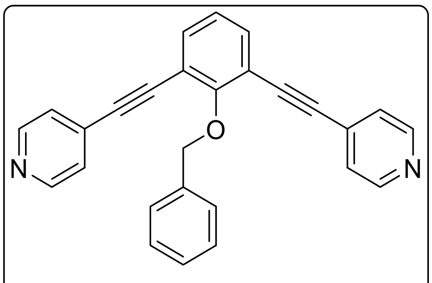 **LBn**	0.01 µmol, 1.0 mol%	1.7×10^{-3} min^{-1}	8.0×10^{-2} mM/min
	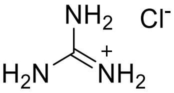 **Guanidine HCl**	0.24 µmol, 24.0 mol%	1.6×10^{-3} min^{-1}	5.2×10^{-2} mM/min

*Three non-zero datapoints were obtained for fitting, as such the error may be larger (R^2 = 0.85, see Figure S6).

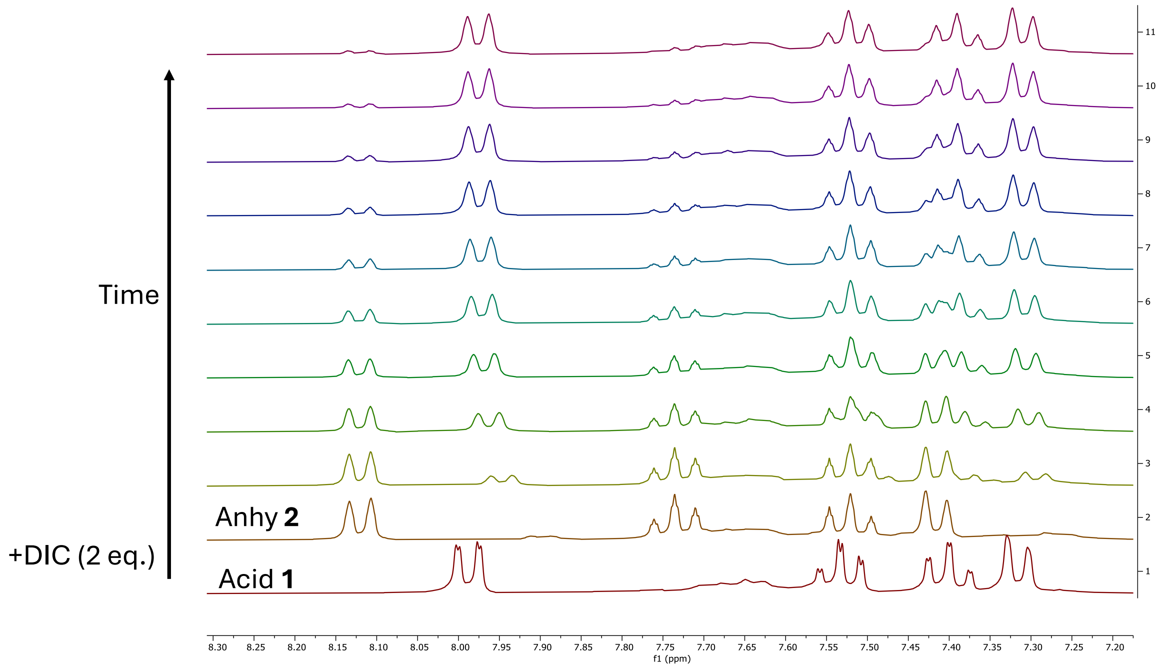

*Figure S1: Partial ^1H-NMR spectra (CD_3CN/D_2O 4/1, 300 MHz, 30°C) of the switch from acid **1** to anhydride **2** upon addition of DIC (2.0 eq.) and the following hydrolysis of the anhydride **2** back to acid **1** all in the presence of $Pt_{12}LGua_{24}$ (1 mol%).*

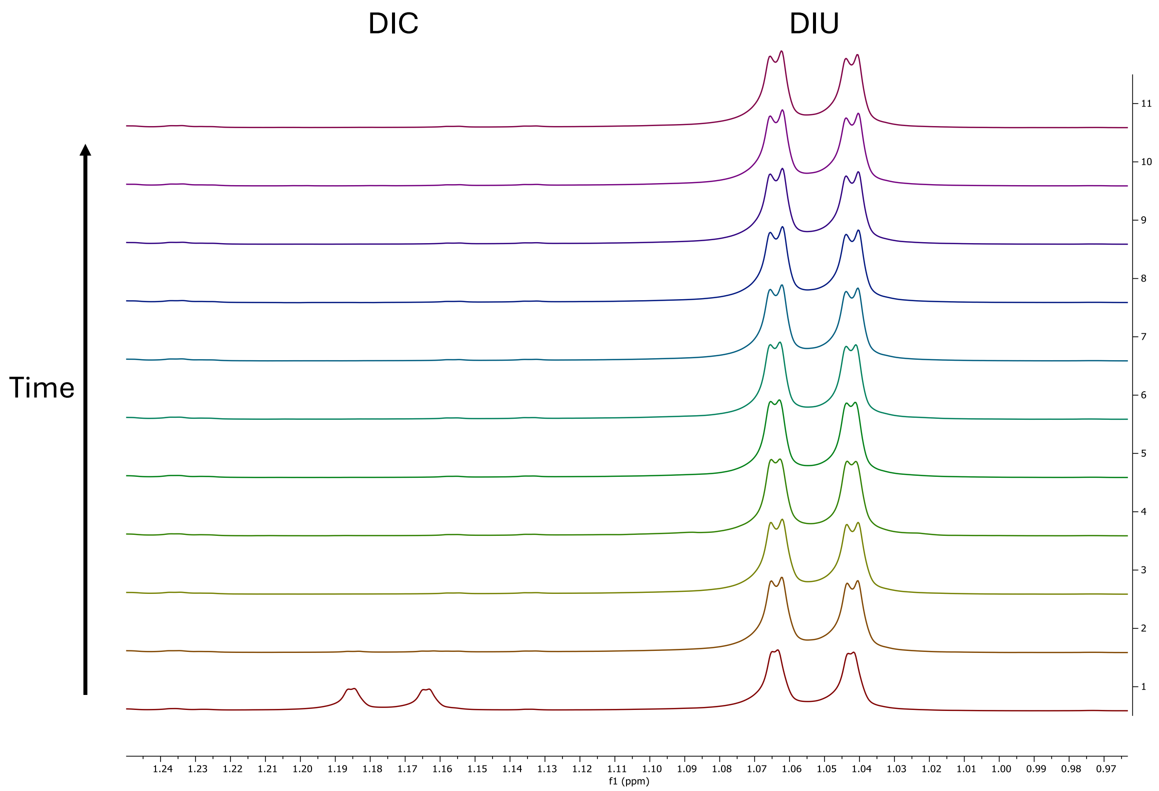

*Figure S2: Partial ^{1}H-NMR spectra (CD_3CN/D_2O 4/1, 300 MHz, 30°C) of the switch from DIC to DIU upon addition of DIC (2.0 eq.) to **1** in the presence of $Pt_{12}LGua_{24}$ (1 mol%).*

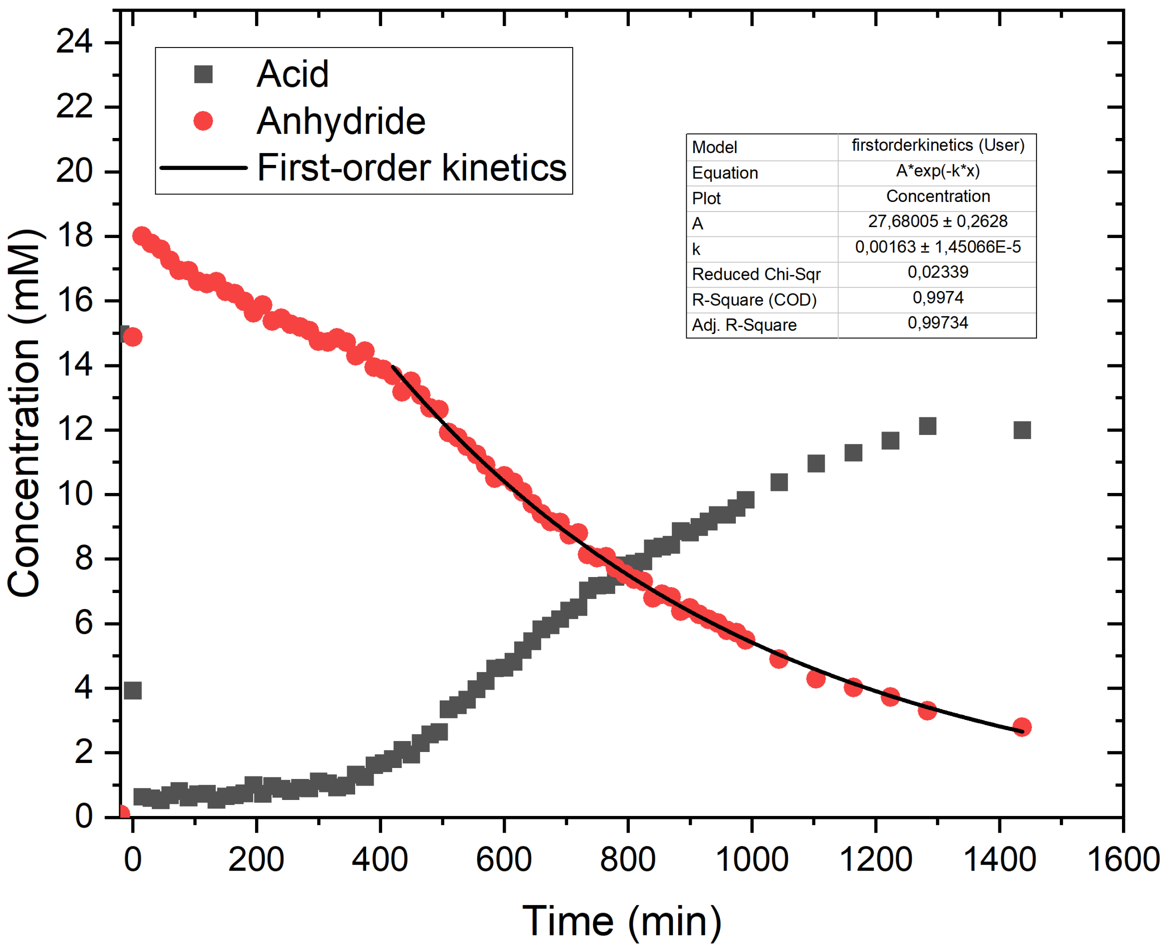

*Figure S3: Reaction profiles of the equilibrium between **1** and **2** agitated by addition of DIC without additive present. First order fit of the final part of the profile. Recovered acid = 61 %, thus selectivity for this cycle is 61%.*

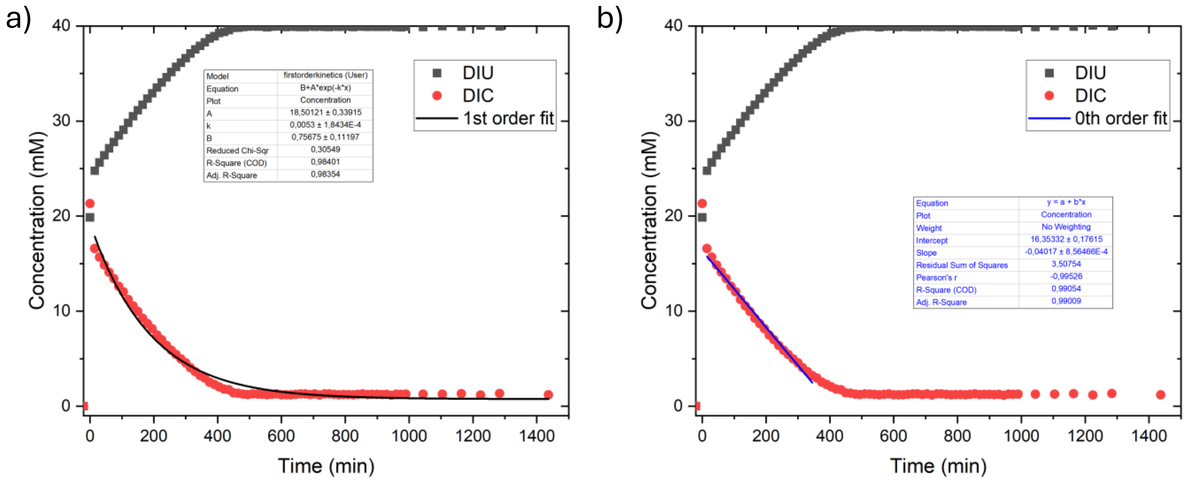

*Figure S4: Reaction profiles of the equilibrium between **1** and **2** agitated by addition of DIC without additive present. (a) 1st order fit (in black and black table) and (b) 0th (in blue and blue table) order fits. A simple order-model may not be sufficient to describe these kinetics.*

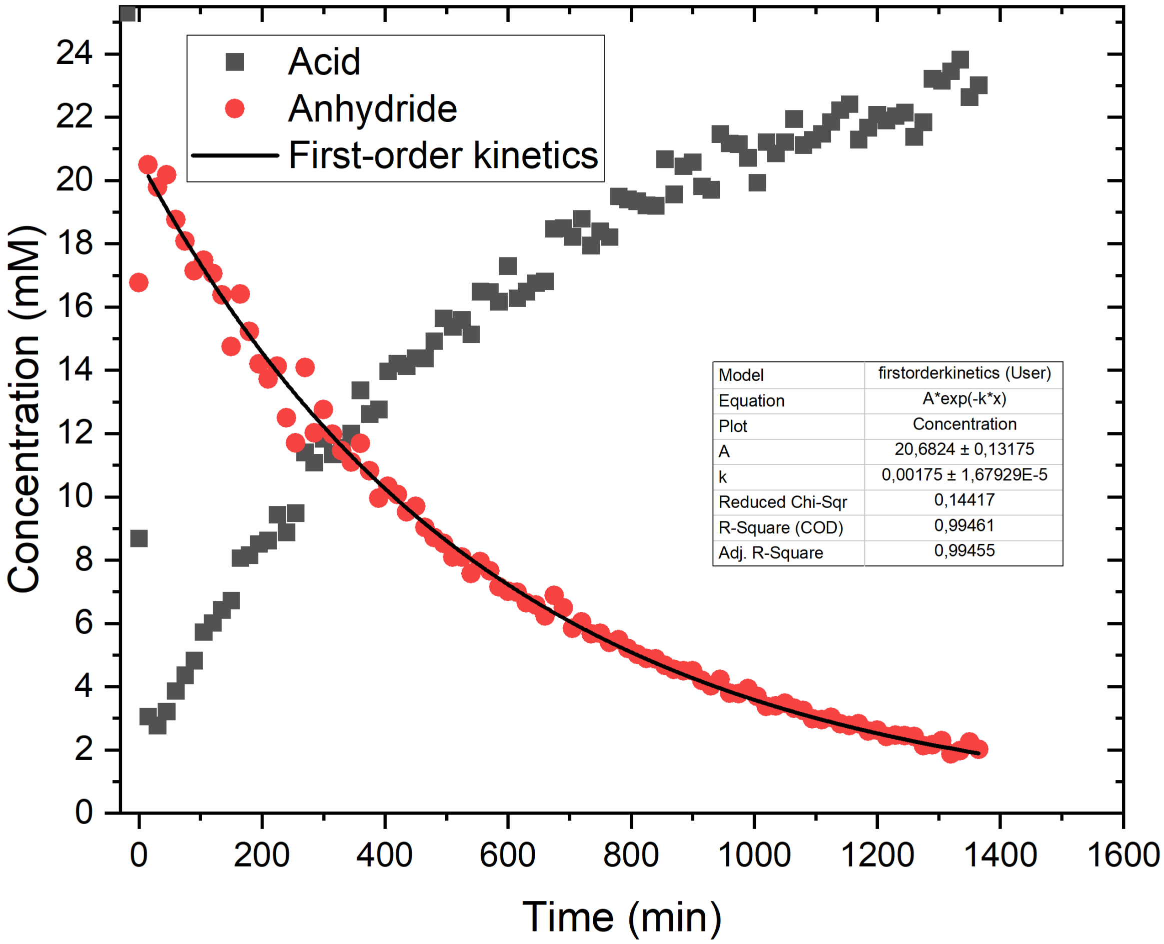

*Figure S5: Reaction profiles of the equilibrium between **1** and **2** agitated by addition of DIC with 1 mol% $Pt_{12}LGua_{24}$ present. First order fit of the final part of the profile. Recovered acid = 93 %, thus selectivity for this cycle is 93%.*

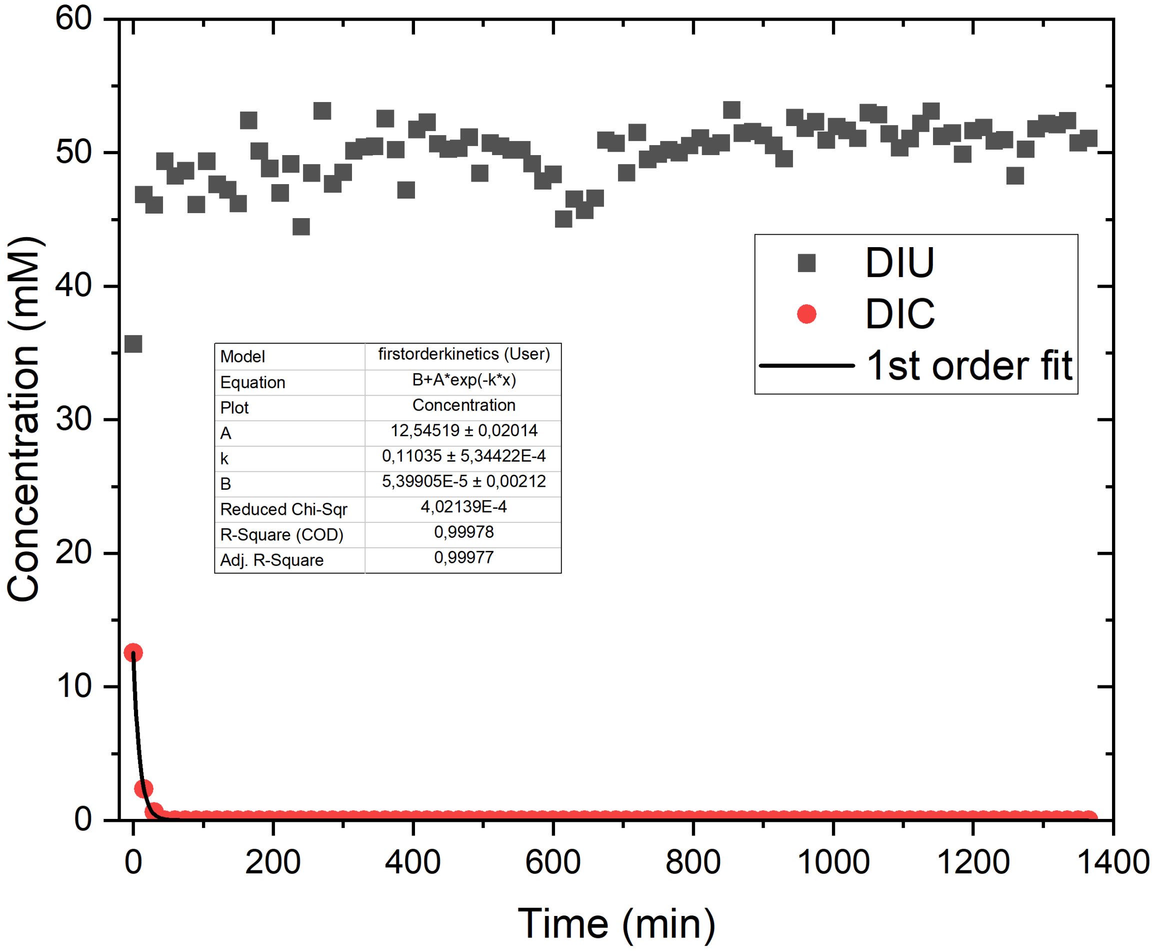

*Figure S6: Reaction profiles of the equilibrium between **1** and **2** agitated by addition of DIC with 1 mol% $Pt_{12}LGua_{24}$ present.*

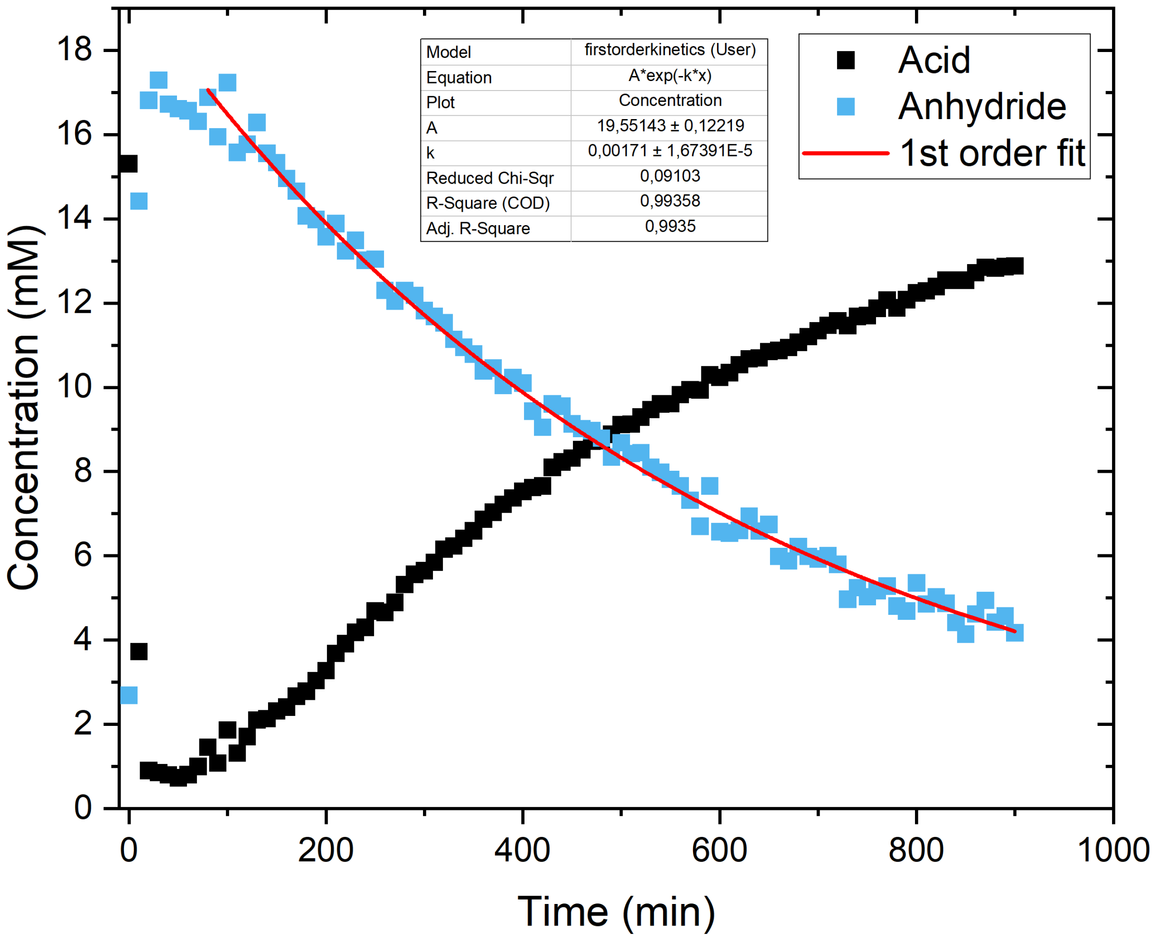

*Figure S7: Reaction profiles of the equilibrium between **1** and **2** agitated by addition of DIC with 1 mol% $Pt_{12}LBn_{24}$ present.*

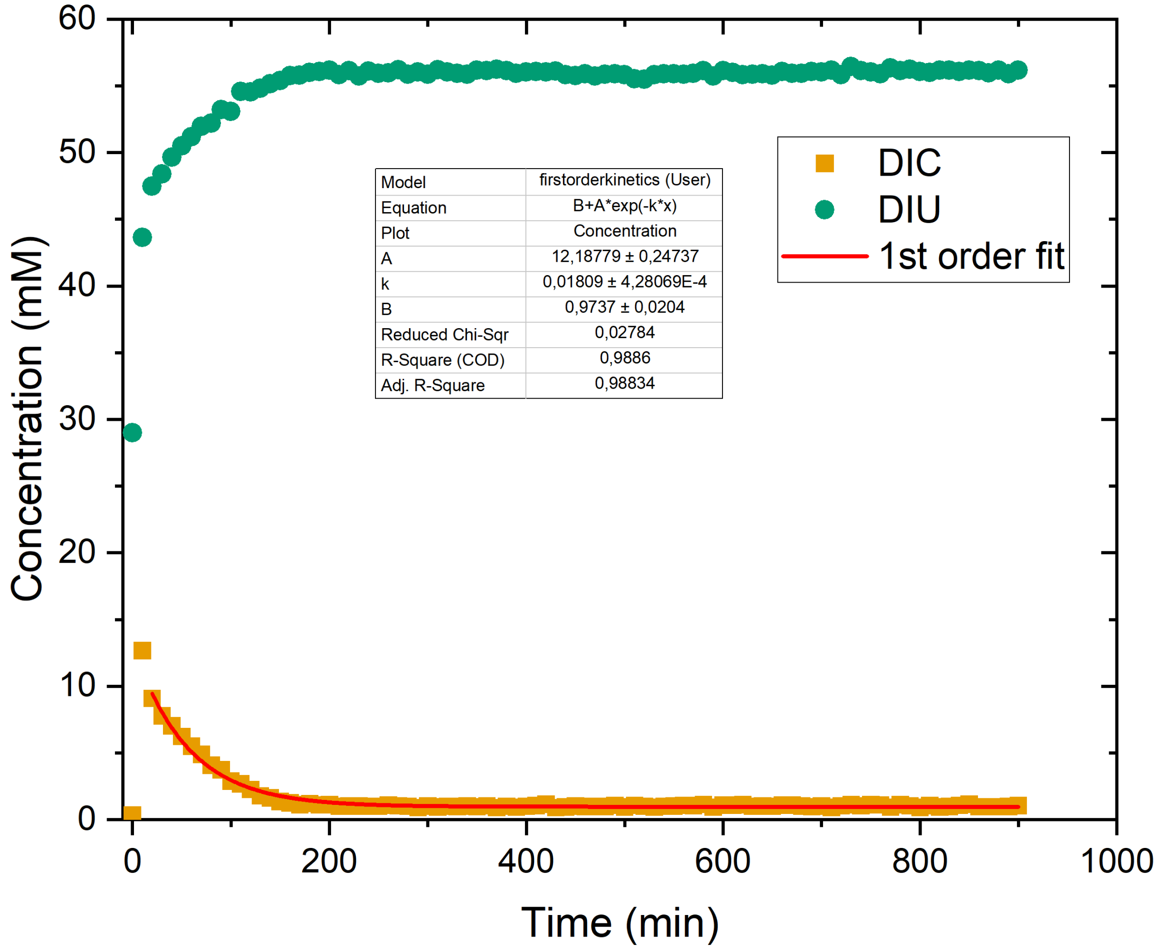

*Figure S8: Reaction profiles of the equilibrium between **1** and **2** agitated by addition of DIC with 1 mol% $Pt_{12}LBn_{24}$ present.*

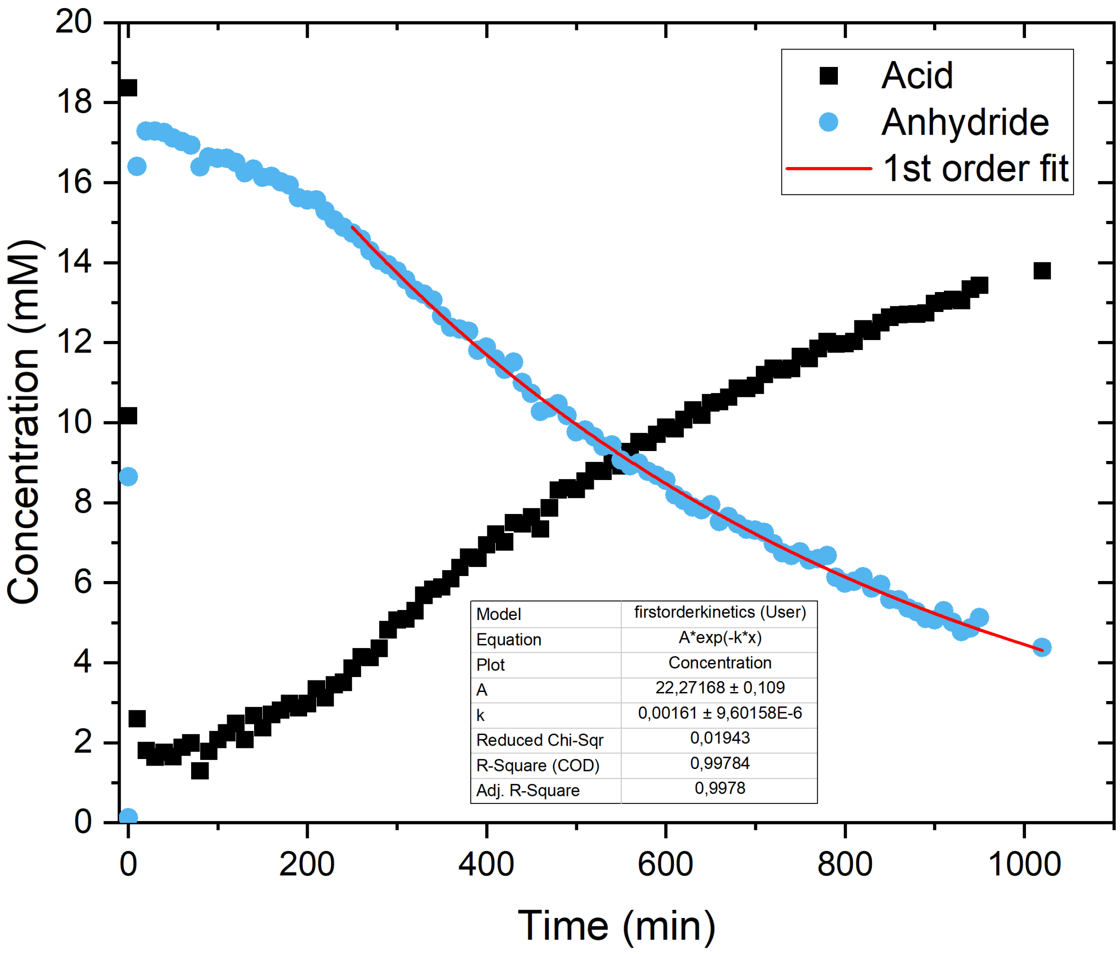

*Figure S9: Reaction profiles of the equilibrium between **1** and **2** agitated by addition of DIC with 24 mol% Guanidine HCl present.*

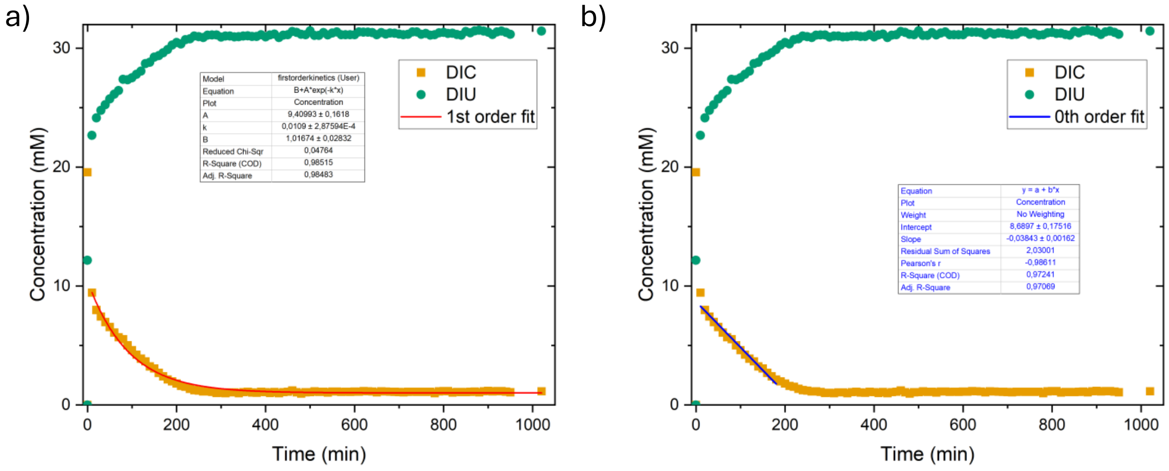

*Figure S10: Reaction profiles of the equilibrium between **1** and **2** agitated by addition of DIC with 24 mol% Guanidine HCl present. With (a) 1st (in black and black table) and (b) 0th (in blue and blue table) order fits are shown. A simple order-model may not be sufficient to describe these kinetics.*

The kinetic fits for the consumption of DIC are not easily and generally described by first or zeroth order kinetics. To get a proper comparison between the experiments with different additives, we fitted the initial rates and used them for comparison.

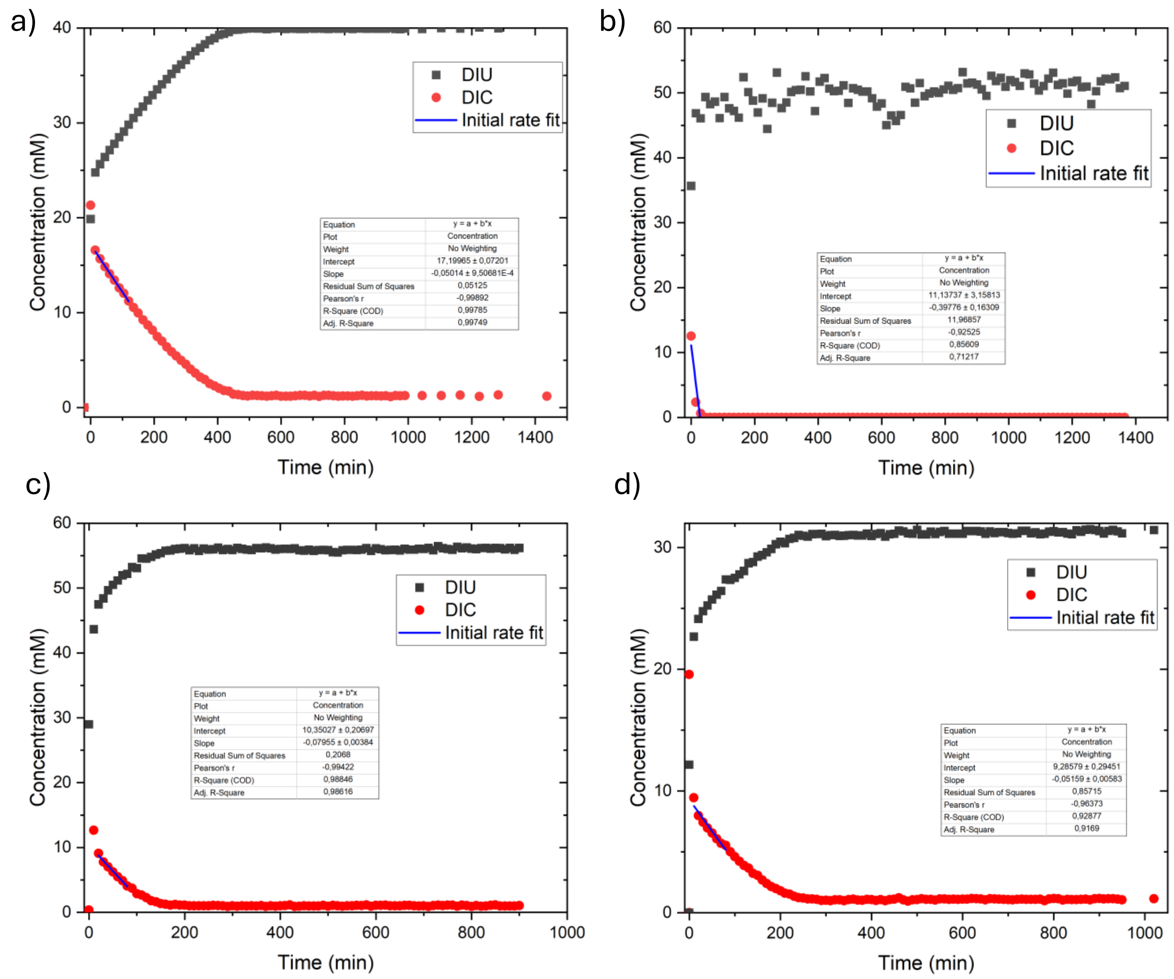

*Figure S11: Reaction profiles of the equilibrium between **1** and **2** agitated by addition of DIC (2.0 eq.) and their corresponding initial rate fits with various additives (a) no additive, (b) 1 mol% $Pt_{12}LGua_{24}$, (c) 1 mol% $Pt_{12}LBn_{24}$, (d) 24 mol% Guanidine HCl.*

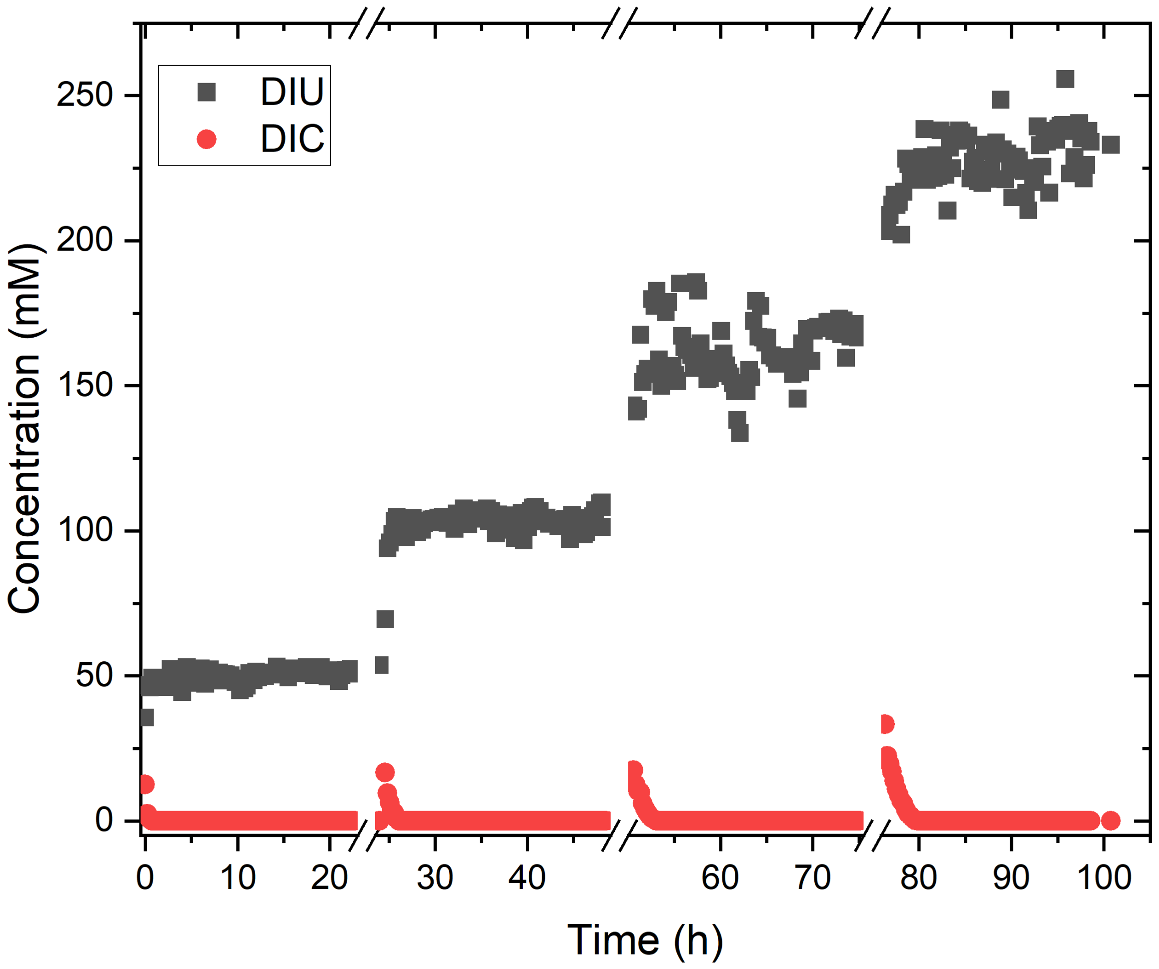

*Figure S12: Reaction profiles of the equilibrium between **1** and **2** agitated by addition of DIC with $Pt_{12}LGua_{24}$ present. Breaks indicate consecutive DIC additions.*

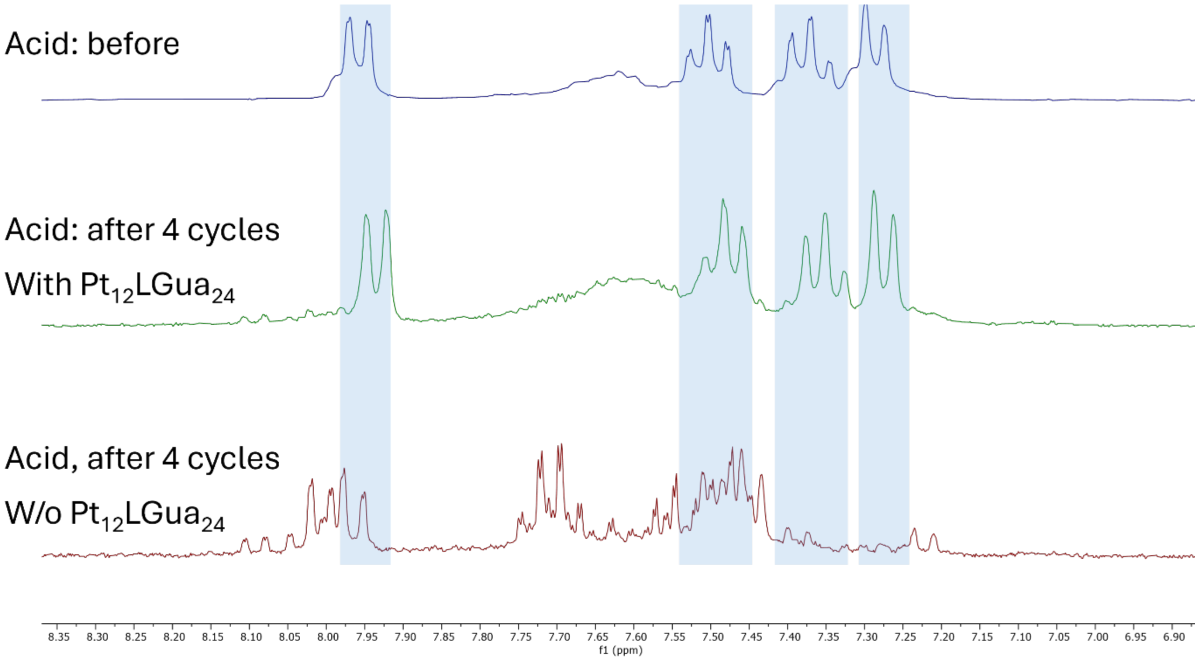

*Figure S13: ^{1}H-NMR spectra of the reaction mixtures after 4 consecutive DIC additions starting with homophthalic acid **1** (20 mM) and with and without $Pt_{12}LGua_{24}$ (1 mol%) in CD_3CN/D_2O (4/1). Resonances belonging to **1** are highlighted in blue. Right three signals are not identifiable without $Pt_{12}LGua_{24}$ (bottom)*

To validate that acid **3** behaves similarly, the same experiments were performed with acid **3** instead of acid **1**. When using acid 3, there is much less side-product formation and as such the selectivity of the cycle improves significantly. However, the DIC hydrolysis follows the same behavior as with acid **1**. Importantly, using acid **3**, the cycle can also undergo multiple additions of DIC without losing much material.

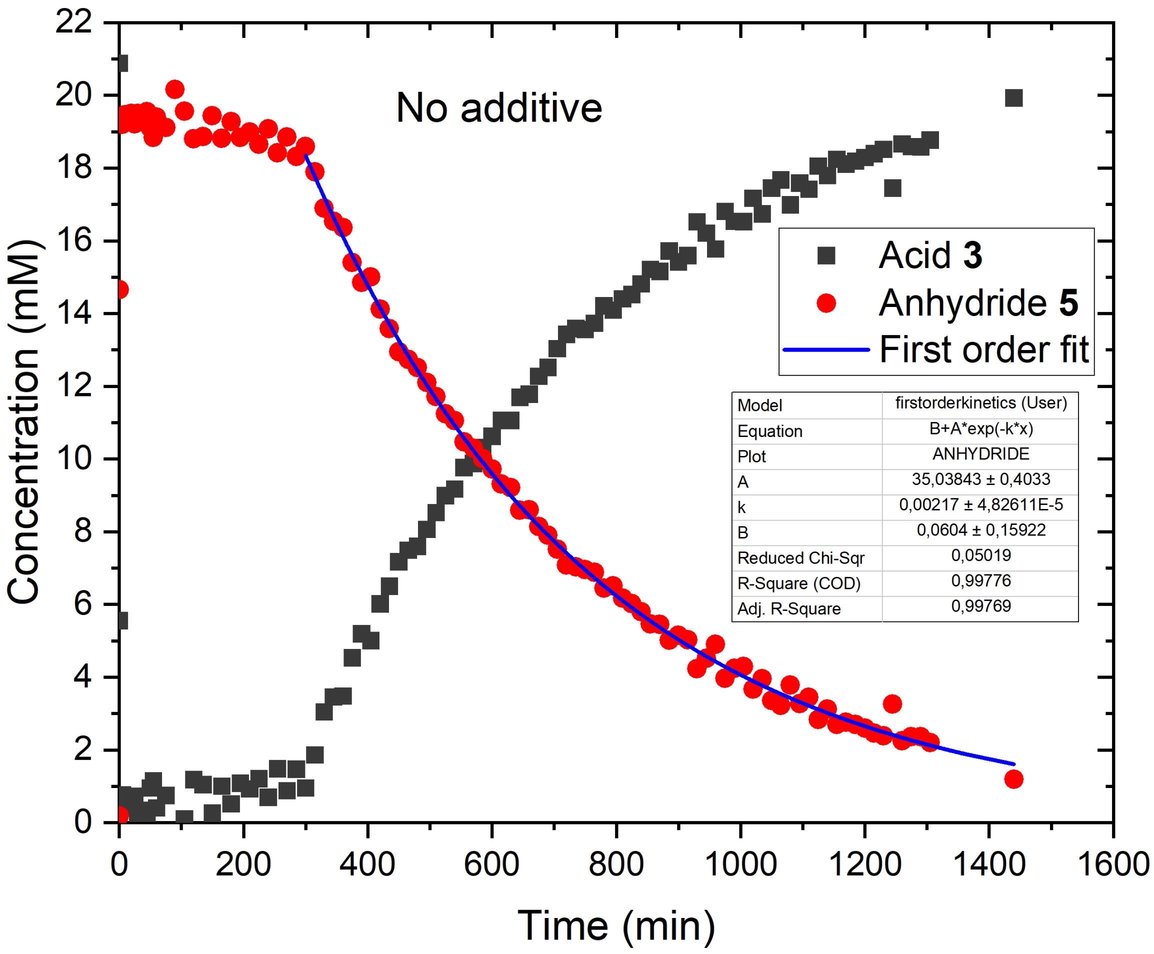

*Figure S14: Reaction profiles of the equilibrium between **3** and **5** agitated by addition of **DIC** (2.0 eq.) without additive present. First order fit of the final part of the profile. Recovered acid = 95 %, thus selectivity for this cycle is 95%.*

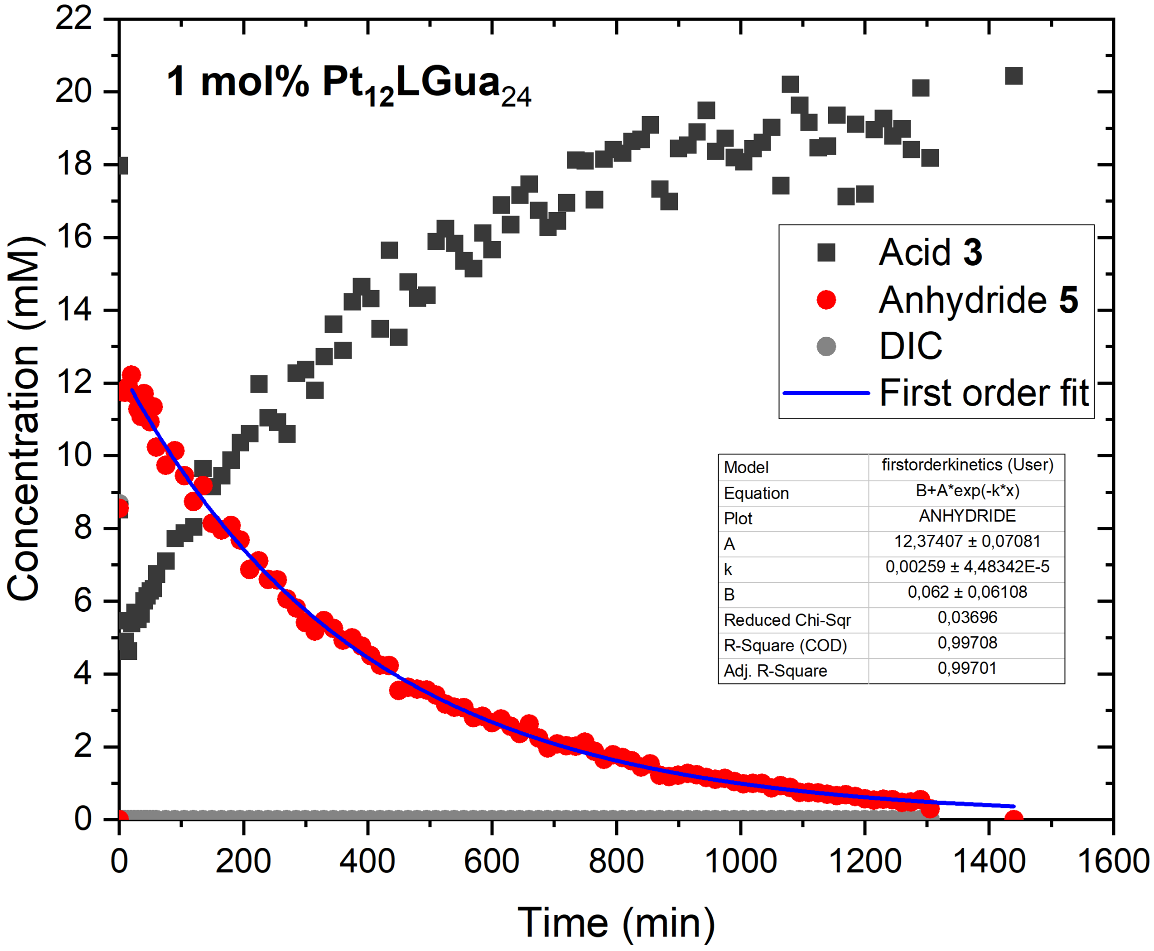

*Figure S15: Reaction profiles of the equilibrium between **3** and **5** agitated by addition of **DIC** (1.5 eq.) with 1 mol% $Pt_{12}LGua_{24}$ present. First order fit of the final part of the profile. Recovered acid = >99%, thus selectivity for this cycle is >99%.*

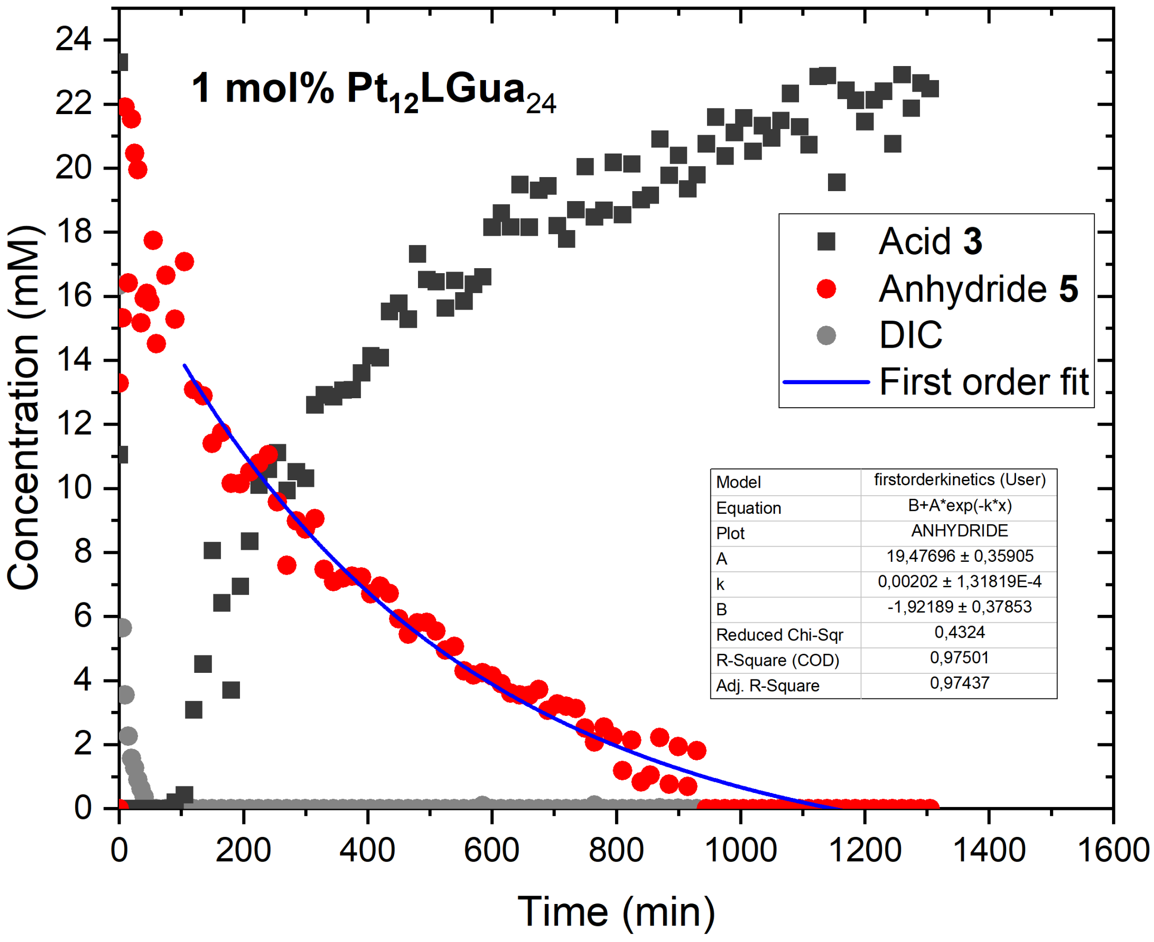

*Figure S16: Reaction profiles of the equilibrium between **3** and **5** agitated by a second addition of **DIC** (2.0 eq.) with 1 mol% **$Pt_{12}LGua_{24}$** present. First order fit of the final part of the profile. Recovered acid = 96%, thus selectivity for this cycle is 96%.*

3. Photocatalytic experiments

General procedure for decarboxylative oxygenation

A stock solution of substrate (2.5 µmol, 1.0 eq.) in CH_3CN and a stock solution of catalyst (25 nmol, 1.0 mol%, 60 µL) in CD_3CN were added together and diluted with CH_3CN and/or H_2O to obtain the desired solvent ratio. In all cases, the final substrate concentration was 5.0 mM. The mixture was stirred in air, irradiated and air-cooled for 16h in the UFO reactor,[7] with a 390 nm Kessil lamp (26 W, 50%), while the temperature remained 27°C. After reaction completion, a stock solution of a known amount of 1,3,5-trimethoxybenzene in CH_3CN was added to the reaction mixture. After shaking, the mixture was analyzed by GC-MS and by FID-integration versus the internal standard, the yield was determined using a calibration curve.

***Table S2:** Control reactions for the decarboxylative oxygenation of **3** to **4**. Conditions are described above.*

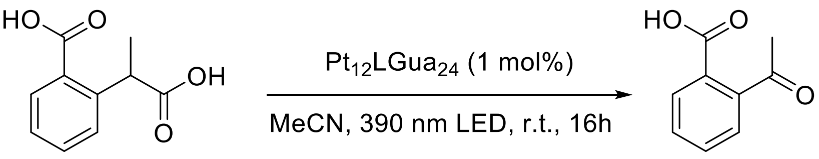

Entry	Deviations	Yield [%][a]
1	-	78
2	No light, 40°C	n.d.
3	No $Pt_{12}LGua_{24}$	n.d.
4	With Et_3N (2.0 eq.)	30
5	Under Argon	12

[a]based on GC integration versus 1,3,5-trimethoxybenzene as external standard. n.d. = not detected

Table S3: *Water dependence for the decarboxylative oxygenation of* ***3*** *to* ***4*** *with* $Pt_{12}LGua_{24}$ *as catalyst. Conditions are described above.*

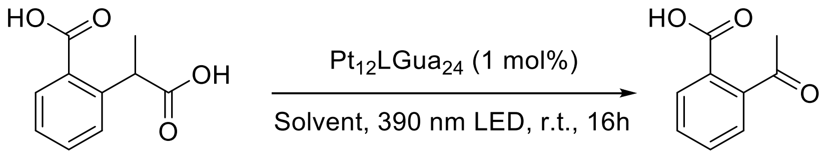

Entry	CH_3CN/H_2O ratio	Water (%)	Yield [%][a]
1	1/0	0	78
2	49/1	2	34
3	19/1	5	27
4	9/1	10	18
5	4/1	20	8

[a]based on GC integration versus 1,3,5-trimethoxybenzene as external standard.

Table S4: *Control reactions for the decarboxylative oxygenation of* ***5*** *to* ***4*** *with* $Pt_{12}LGua_{24}$ *as catalyst. Conditions are described above.*

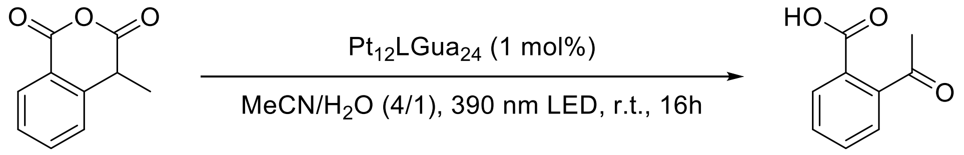

Entry	Deviations	Yield [%][a]
1	-	86
2	No light, 40°C	n.d.
3	No $Pt_{12}LGua_{24}$	n.d.
4	No H_2O	70

5	Under Argon	16
6	With NaN_3 (3.0 eq.)	n.d.

[a]based on GC integration versus 1,3,5-trimethoxybenzene as external standard. n.d. = not detected

Table S5: *Photocatalyst screen for the decarboxylative oxygenation of **5** to **4**. Conditions are described above.*

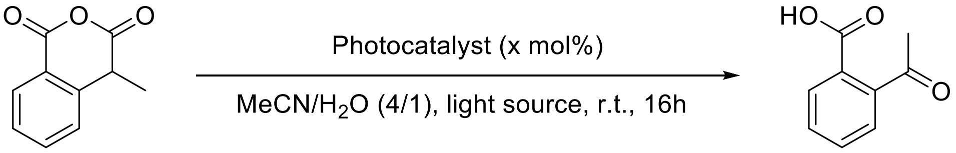

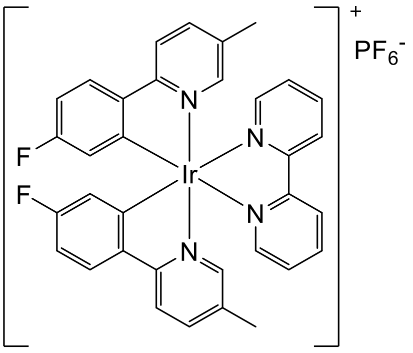

Ir(F(Me)ppy)$_2$(bpy)PF$_6$

Entry	Photocatalyst [mol%]	Light source	Yield [%][a]
1	$Pt_{12}LGua_{24}$ [1.0]	390 nm	86
2	$Pt_{12}LBn_{24}$ [1.0]	390 nm	64
3	Ir(F(Me)ppy)2(bpy)PF6 [9.2]	456 nm	72
4	Methylene Blue [10.0]	640 nm	72

[a]based on GC integration versus 1,3,5-trimethoxybenzene as external standard.

4. Mechanistic investigations

The decarboxylative oxygenation of 2-phenylpropionic acid to acetophenone was selected as model to test the reactivity in buffered medium as we had experience with this reaction.[3] As buffer, 2-(*N*-morpholino)ethanesulfonic acid (MES) was dissolved in D_2O (0.05 M), which was adjusted to pH_{obs} = 5.3 with NaOD in D_2O (1.0 M). Then, this solution was added as described by the general procedure, substituting the H_2O addition for buffer addition. This gave a MeCN/D_2O (4/1) mixture including 100 mM MES. The rest of the general procedure described above in section 3 was followed.

Table S6*: Decarboxylation of 2-phenyl propionic acid to acetophenone in buffered solutions.*

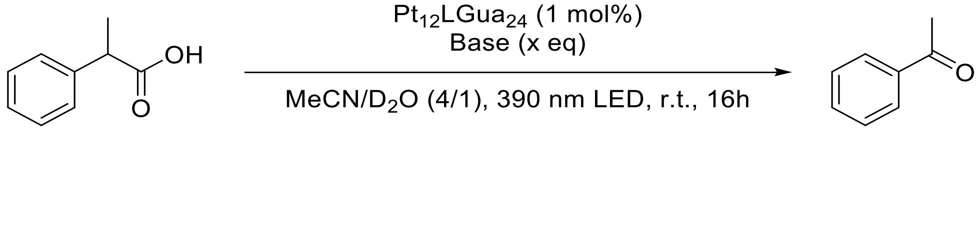

Entry	Base	Buffered	Yield [%][a]
1	None	MES 100 mM (pH_{obs}= 5.3)	n.d.
2	NaOH (2.0 eq)	MES 100 mM (pH_{obs}= 5.3)	n.d.
3	Et_3N (2.0 eq)	MES 100 mM (pH_{obs}= 5.3)	n.d.
4	None	-	3
5	NaOH (4.0 eq)	-	50
6	Et_3N (2.0 eq)	-	42

[a]based on GC integration versus 1,3,5-trimethoxybenzene as external standard. n.d. = not detected

UV-Vis titrations

To study if there are interactions between either substrate and the nanosphere, UV-Vis titrations were carried out.

Spectra were recorded at 20°C. Briefly, a solution of $Pt_{12}LGua_{24}$ (0.4167 µM) was prepared in the desired solvent (4 mL total). For the acid: MeCN/H2O 4/1 and for the anhydride: MeCN. The anhydride hydrolyses in water and as such we decided to use just MeCN. Of the host solution, 3 mL was added to a quartz cuvette and a UV-Vis spectrum was recorded against a solvent reference cuvette. Then, a stock solution of acid or anhydride was added to the cuvette containing the host and the UV-Vis spectrum was recorded again, which was repeated to obtain the equivalents of guest/host shown in the figures.

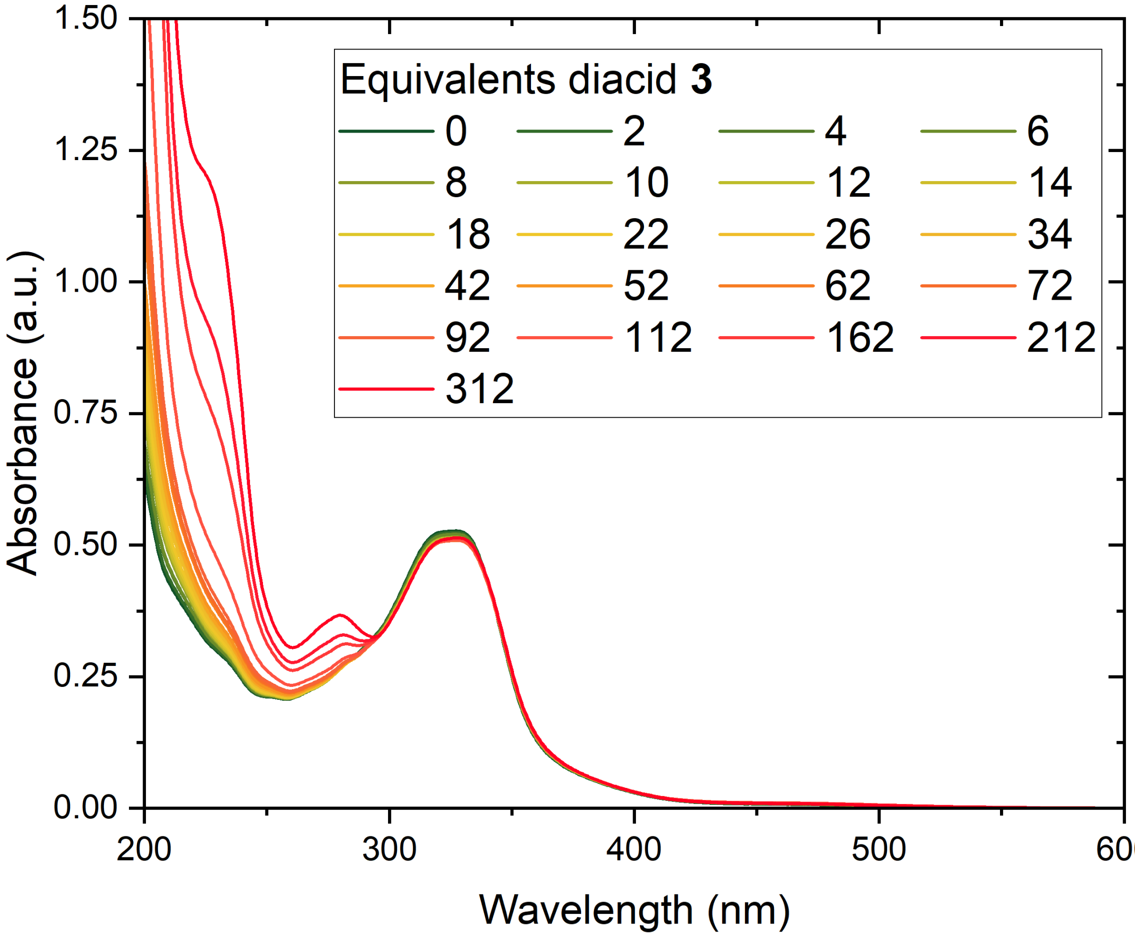

*Figure S17: UV-Vis spectra of $Pt_{12}LGua_{24}$ (starting concentration: 0.4167 µM) upon gradual addition of methyl-homophthalic acid **3** in CH_3CN/H_2O (4/1). The main cage absorption peak (maximum at 325 nm) does not change upon addition of guest. The slight decrease is due to a dilution effect.*

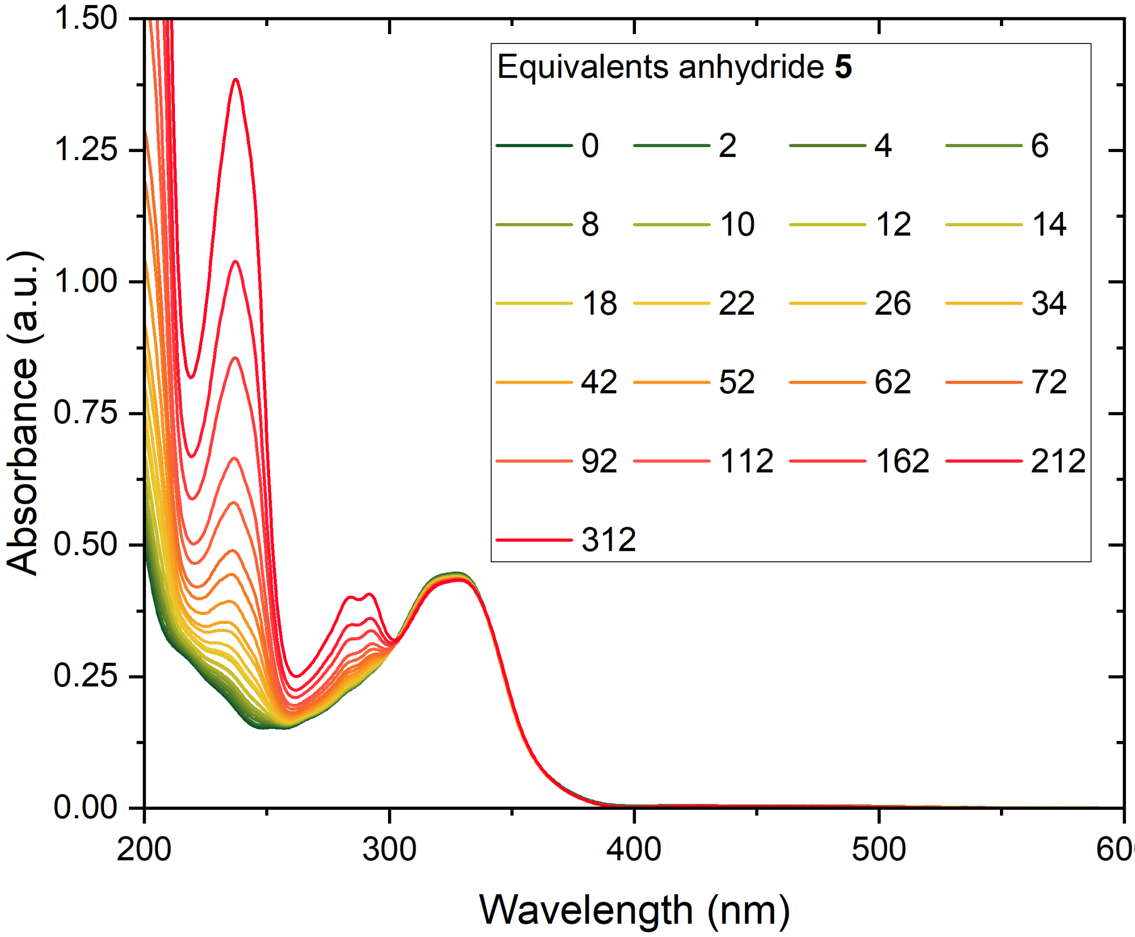

*Figure S18: UV-Vis spectra of $Pt_{12}LGua_{24}$ (starting concentration: 0.4167 µM) upon gradual addition of methyl-homophthalic anhydride **5** in pure CH_3CN. The main cage absorption peak (maximum at 325 nm) does not change upon addition of guest. The slight decrease is due to a dilution effect.*

EPR experiments

A solution of 0.1 M $TBAPF_6$ (20.3 mg) in CH_3CN (0.5 mL) containing methyl-homophthalic anhydride **5** (0.44 mg, 2.5 µmol, 1.0 eq.) $Pt_{12}LGua_{24}$ (25 nmol, 1 mol%). The solution was shaken and added to a J-young EPR tube. The liquid was slowly frozen with liquid nitrogen and cooled further with liquid He in the EPR machine to 10 Kelvin. Spectra were first taken in the dark. Then, the EPR tube was illuminated with a 390-500 nm bandwidth lamp (Honle) for 1 minute and the spectrum was recorded under illumination.

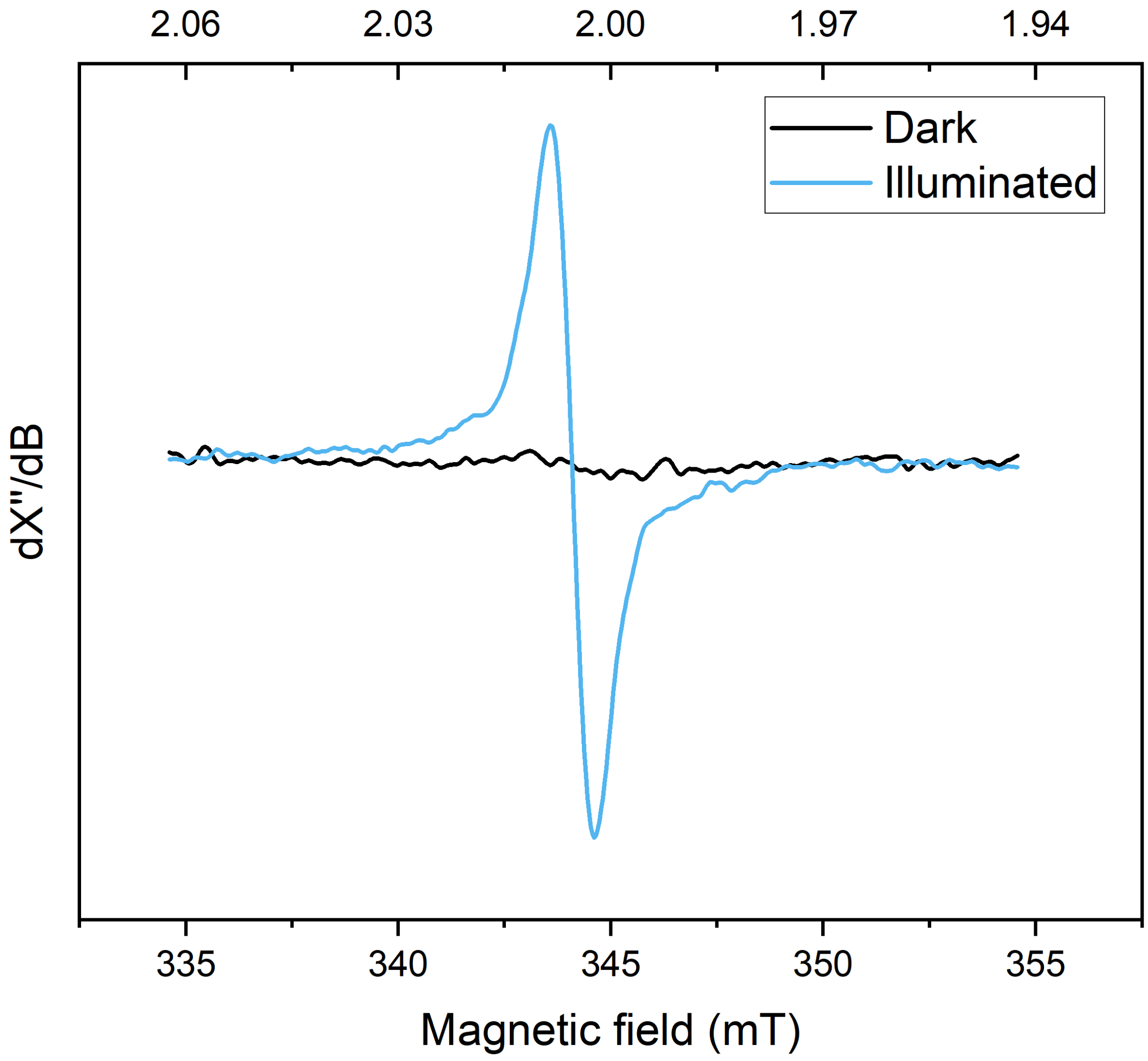

*Figure S19: EPR spectra of the mixture of anhydride **5** and $Pt_{12}LGua_{24}$ in CH_3CN at 10K as described above. In the dark (black line) and under illumination with a 390-500 nm bandwidth lamp (blue line). Signal is centered at g=2.003, indicating an organic radical. Settings (dark): 9.646771 GHz, 2.000 mW, modulation amplitude: 4 G. Settings (illuminated): 9.646087 GHz, 2.000 mW, modulation amplitude: 4 G.*

Stern-Volmer quenching studies

All the quenching studies were conducted under aerobic conditions and all spectra were recorded at 20°C. Briefly, a solution of $Pt_{12}LGua_{24}$ (0.4167 µM) was prepared in the desired solvent (4 mL total). Of that solution, 3 mL was added to a quartz cuvette and the steady-state emission spectrum was recorded. Excitation wavelength: 370 nm (5.0 nm bandwidth), emission bandwidth 1.0 nm at 20.0°C. Then, a stock solution of quencher was added to the cuvette containing the host and the emission spectrum was recorded again. The maximum emission intensity was determined for each point and the maximum emission of the first point (containing only the host) was divided by the maximum intensity of each point (I_0/I) and plotted against the quencher concentration.

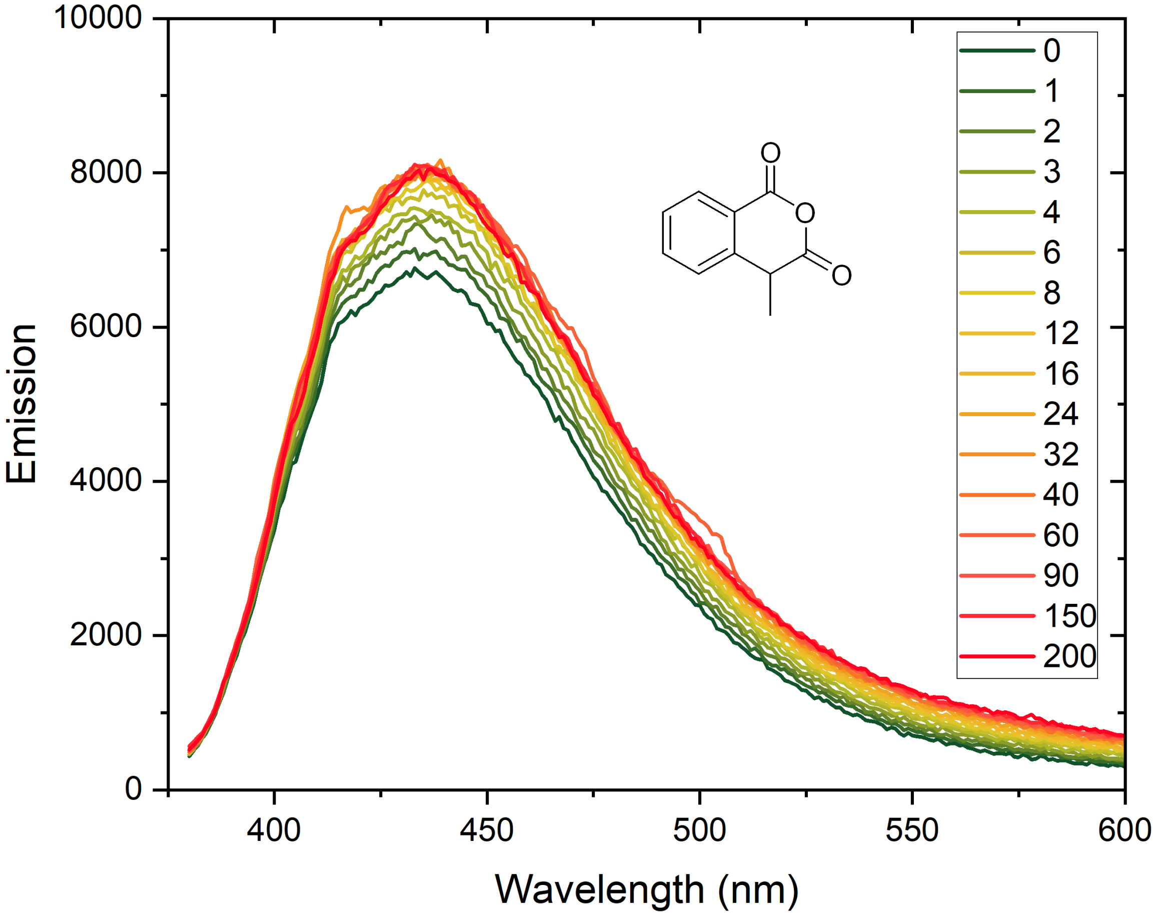

*Figure S20: Steady state emission spectra of $Pt_{12}LGua_{24}$ (starting concentration: 0.4167 µM) upon gradual addition of methyl-homophthalic anhydride **5** in pure CH_3CN. Interestingly, the emission increases slightly, which we attribute to reactivity of the anhydride with reactive oxygen.*

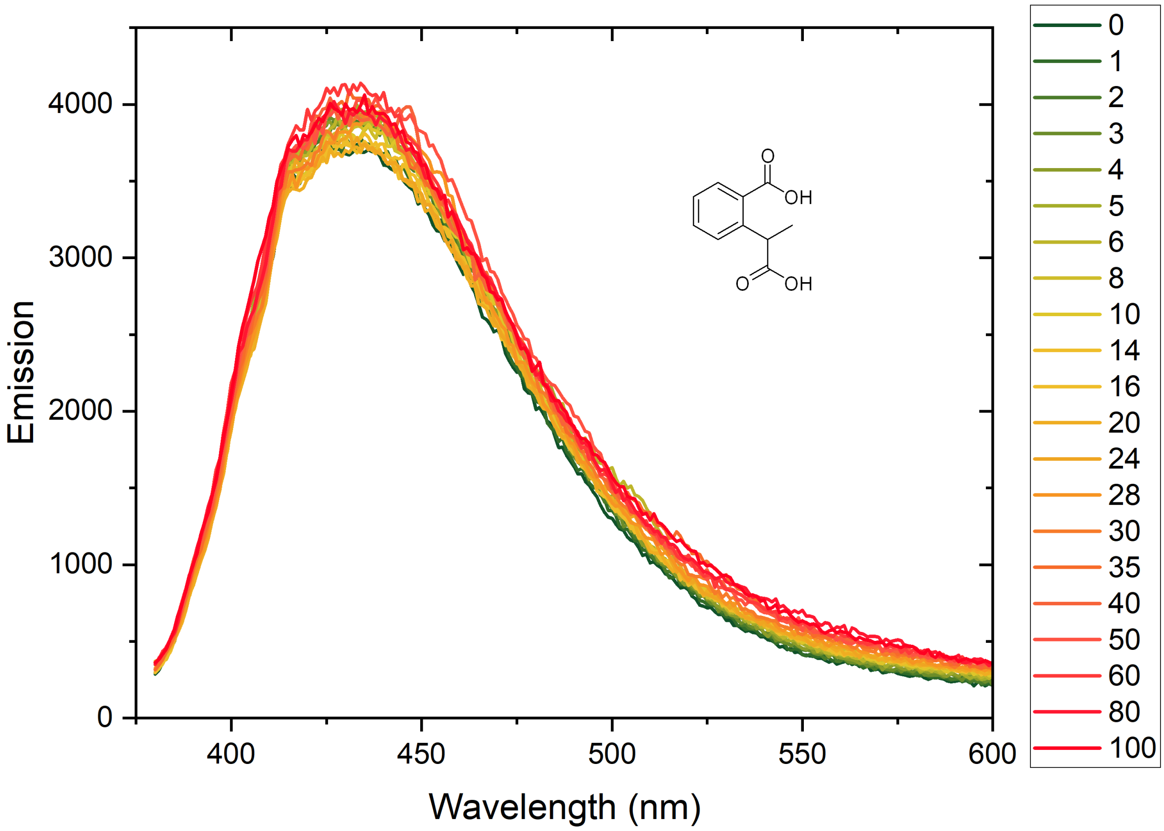

*Figure S21: Steady state emission spectra of $Pt_{12}LGua_{24}$ (starting concentration: 0.4167 µM) upon gradual addition of methyl-homophthalic acid **3** in CH_3CN/H_2O (4/1). The emission intensity is lower than in pure CH_3CN, which we attribute to some aggregation of the nanospheres as H_2O is added.*

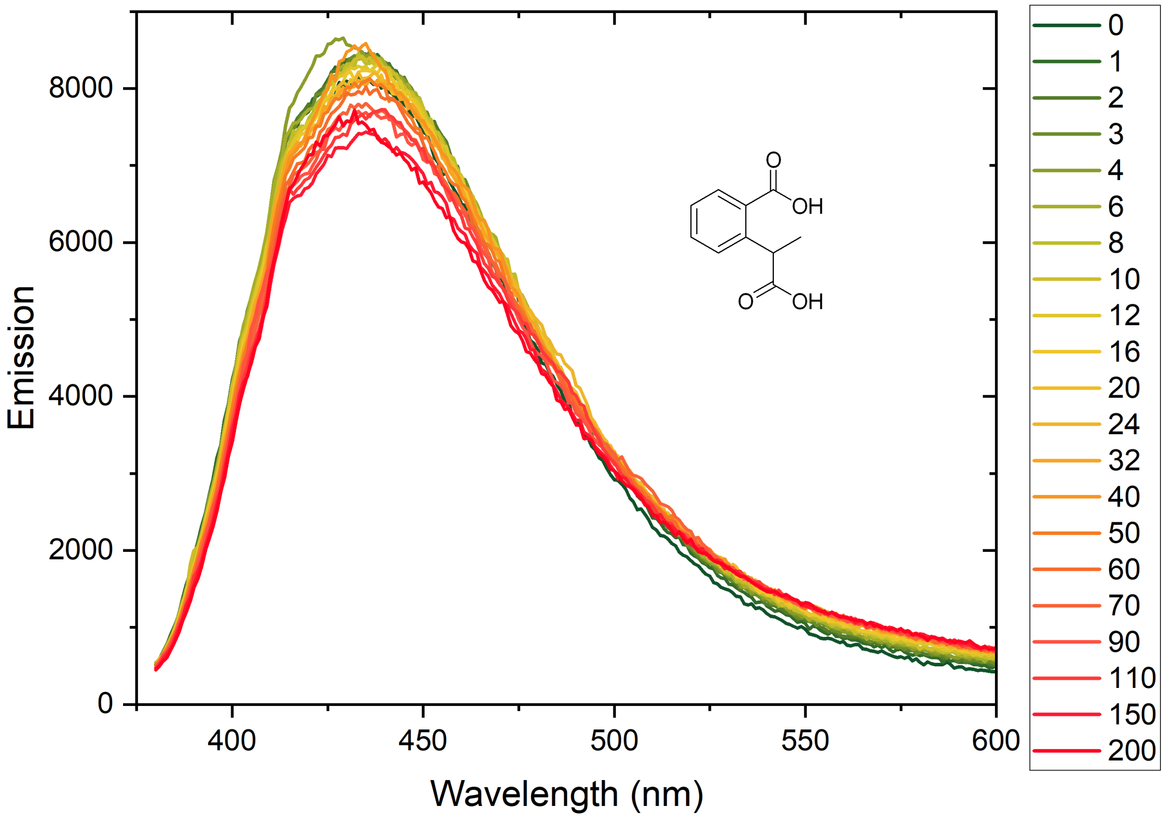

*Figure S22: Steady state emission spectra of $Pt_{12}LGua_{24}$ (starting concentration: 0.4167 µM) upon gradual addition of methyl-homophthalic acid **3** in pure CH_3CN.*

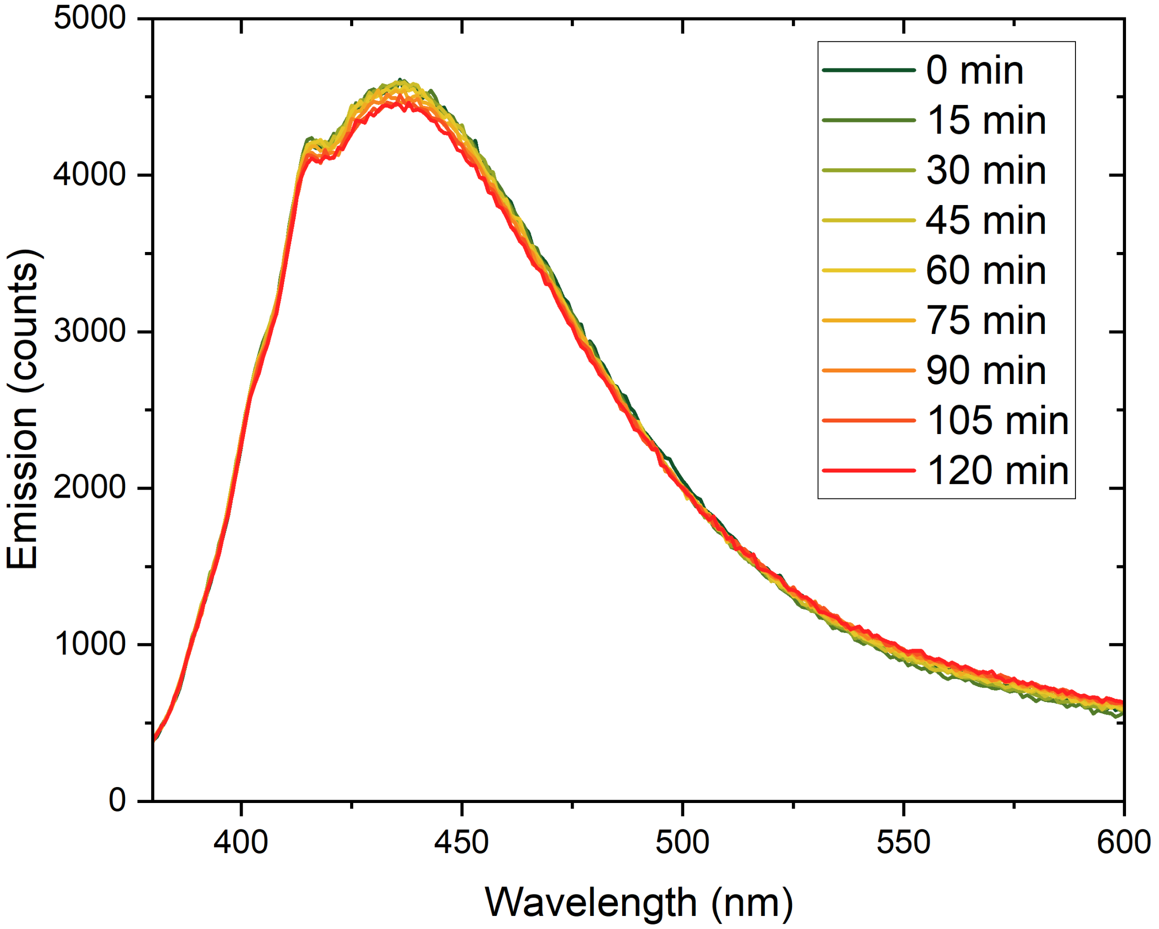

Figure S23: Steady state emission spectra of $Pt_{12}LGua_{24}$ (starting concentration: 0.4167 µM) in CH_3CN/H_2O 4/1 over time. Same settings were applied as for the Stern-Volmer titrations. From these spectra it can be concluded that $Pt_{12}LGua_{24}$ is stable within the timeframe of the Stern-Volmer titrations.

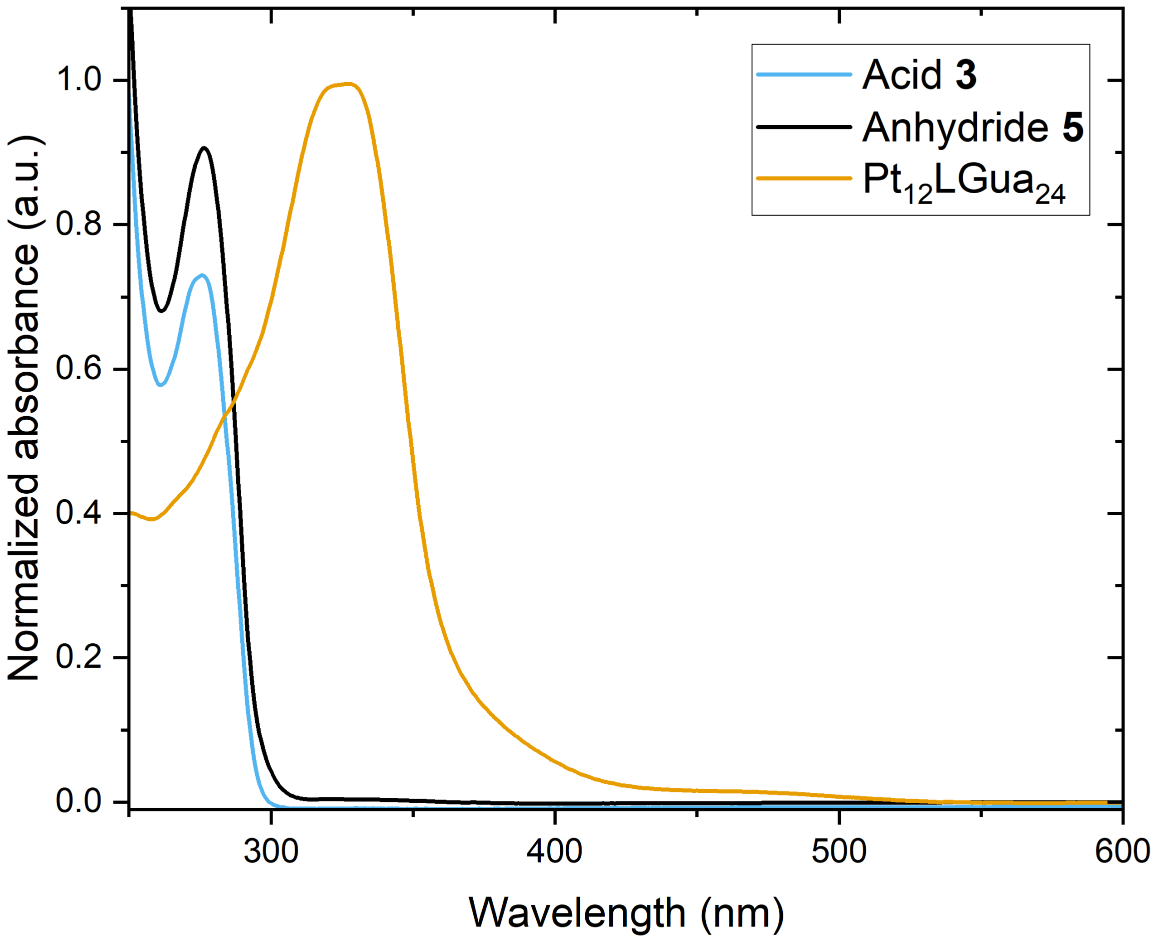

Figure S24: UV-Vis spectra of the molecules used for SV quenching in CH_3CN/H_2O (4/1). Spectra are normalized have a better comparison. At the excitation wavelength used for SV quenching (370 nm) only $Pt_{12}LGua_{24}$ has noticeable absorption.

TEMPO trapping by GC-FI

To the general catalytic mixture as described in ESI section 3, TEMPO (1.95 mg, 12.5 µmol, 5.0 eq.) was added. After reaction, IS was not added, but the mixture was subjected to GC-FI, giving the MS spectrum of the trapped TEMPO adduct below, which is stemming from acetonitrile solvent.

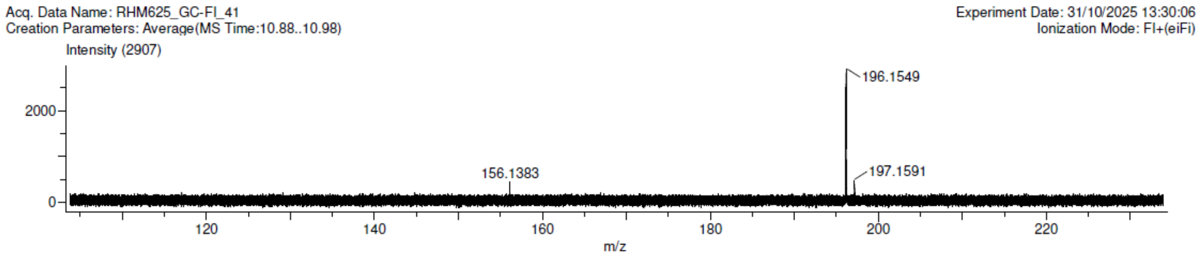

Figure S25: Experimental GC-FI mass spectrum of the TEMPO trapped adduct.

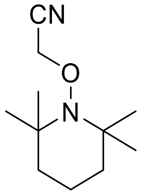

Chemical Formula: $C_{11}H_{20}N_2O$
Calculated Mass: 196.1576
Found Mass: 196.1549

Figure S26: TEMPO trapped adduct in Figure S25 with found and calculated masses.

Gas formation detection

A stock solution of methyl-homophthalic anhydride **5** (5.0 µmol, 1.0 eq.) in CH_3CN and a stock solution of catalyst (50 nmol, 1.0 mol%, 120 µL) in CD_3CN were added together and diluted with CH_3CN to obtain a final substrate concentration of 5.0 mM in 1.0 mL solvent. The vial was septum capped and connected to a inline gas GC via needle and tubing. Then it was irradiated and air-cooled for 30 min in the UFO reactor,[7] with a 390 nm Kessil lamp (52 W, 100%), while the headspace was measured every 2.5 minutes by gas GC. Afterwards, a stock solution of a known amount of 1,3,5-trimethoxybenzene in CH_3CN was added to the reaction mixture. After shaking, the mixture was analyzed by GC-MS and by FID-integration versus the internal standard, the yield was determined to be **12%** using a calibration curve.

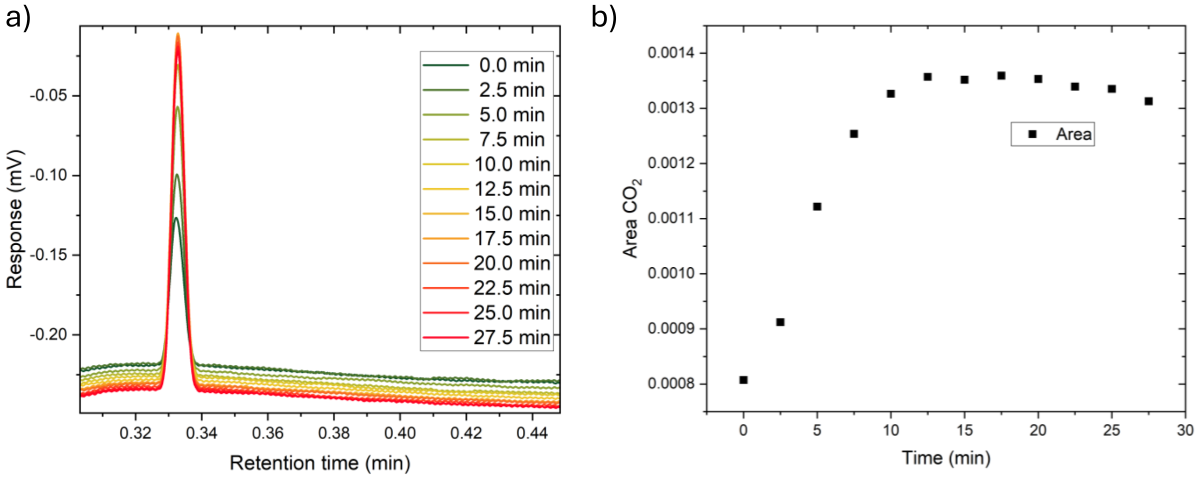

Figure S27: (a) partial gas chromatographs in time from the experiment described above, zoomed in on the retention of CO_2. (b) Integrated area of the CO_2 retention peak over time (integrated between: 0.3255-0.3395 min).

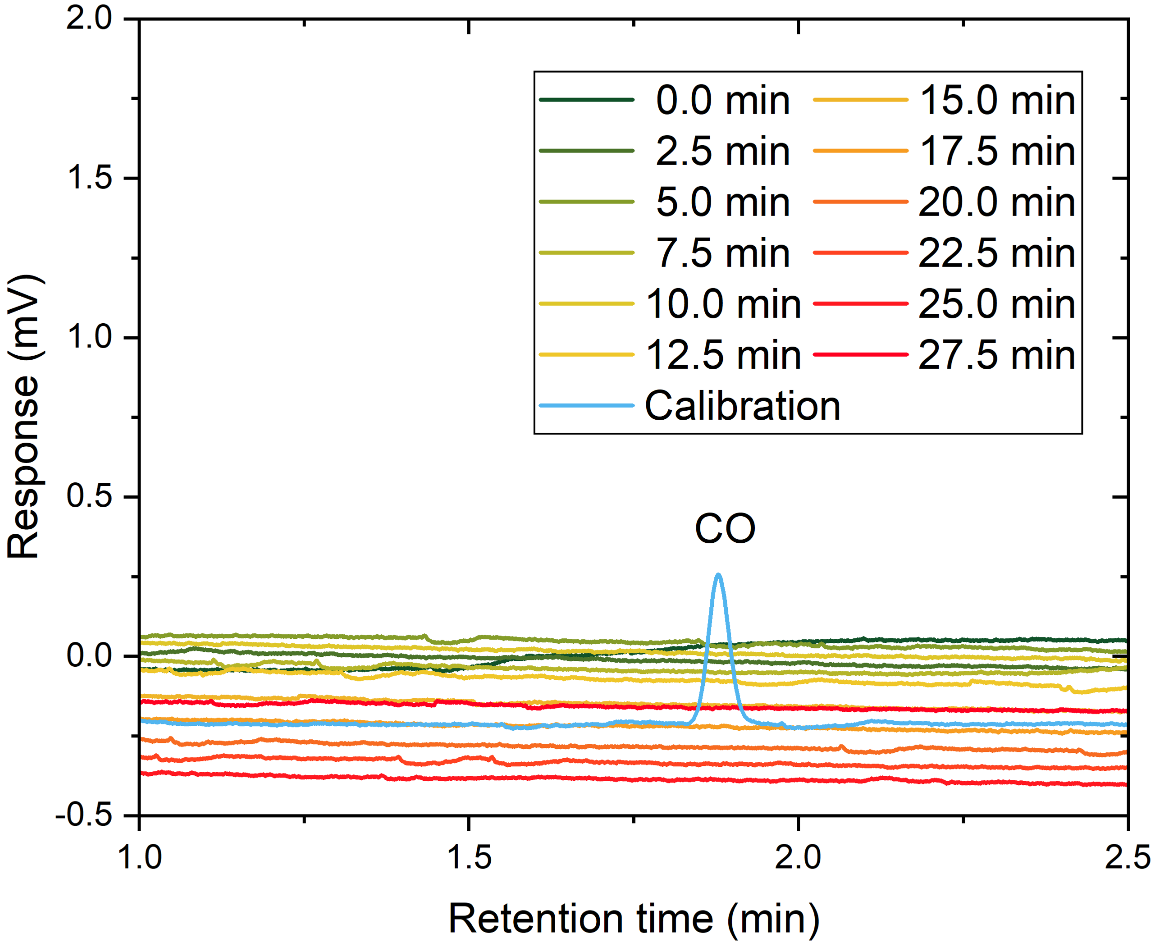

Figure S28: Partial gas chromatographs in time from the experiment described above, zoomed in on the retention of CO, including a calibration gas run to show the retention time for CO.

Due to the nature of the set-up and because the quantities of CO_2 detected in the experiment were outside of the range of the calibration curve, absolute ppms of CO_2 could not be determined

The area of CO_2 increases in the first 10 minutes and then plateaus, whereas no CO was detected. Therefore we conclude that the reaction with anhydride **5** is a decarboxylation reaction which produces CO_2.

We do not observe reductive quenching in CH_3CN/H_2O (4/1), and as such we hypothesized that oxygen might play a crucial role under these conditions in the observed decarboxylation reactivity of anhydride **5**. Further evidence for this was obtained by adding NaN_3 as known $^1O_2$ quencher, which indeed halted conversion. Additionally, when other photocatalysts (Table S5) were used that are well known singlet oxygen generators, the reactivity was still present. That shows that $^1O_2$ is the key reactive intermediate with anhydride **5** to make ketone product **4**.

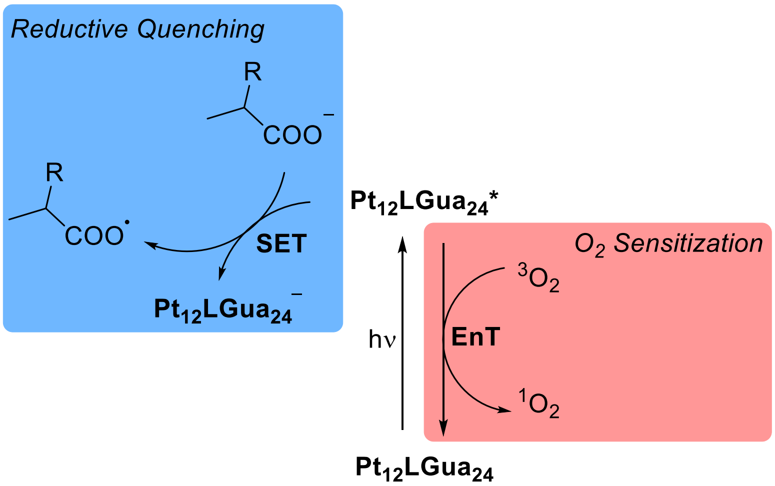

Figure S29: Proposed photochemical mechanisms for reductive quenching via single electron transfer (SET) versus O_2 sensitization via energy transfer (EnT).

5. Online FT-IR spectroscopy

For the FT-IR experiments, a setup was built which is shown in Figure S30. Briefly, a 20 mL vial containing a stir bar and the reaction mixture was setup capped with the in- and outlet of tubing passing through the septum. The tubing were connected to a peristaltic pump and the FT-IR transmission cell such that the reaction mixture would pass via the pump through the transmission cell and back into the reaction mixture. A stirring plate was put underneath the reaction vial and the light source (Thorlabs, CHROLIS-C2 and LLG03-4H light guide, 420 nm with 14 nm bandwidth, 20 % intensity = 142 mW) set 4 cm from the reaction vial wall. The flow rate on the peristaltic pump was set as 0.5 mL/min and the dead volume of the liquid system (inner volume of all the tubing and cell) were about 2.5 mL, *i.e.* a complete liquid exchanged was achieved in 5 minutes. The FT-IR transmission cell had an pathlength of 200 µm and the flow in was connected to the bottom of the cell, flow out was connected to the top. Care was taken that the reaction mixture had plenty of volume that there was continuous flow.

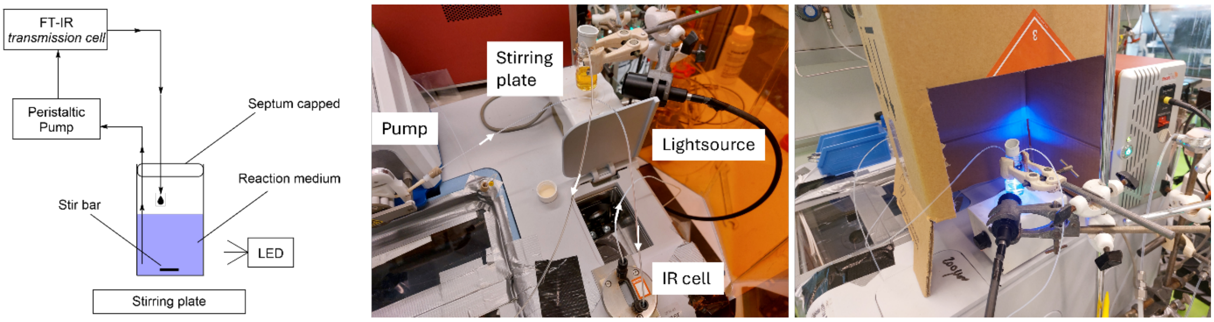

Figure S30: Schematic setup of the FT-IR experiments.

General protocol FT-IR spectroscopy

A stock solution of substrate (42.0 µmol, 1.0 eq.) in CD_3CN and a stock solution of $Pt_{12}LGua_{24}$ (1.0 mol%, 1.0 mL) in CD_3CN were added to a 20 mL vial containing a stir bar and diluted with CD_3CN and D_2O to obtain a 4/1 CD_3CN/D_2O ratio (final volume = 8.33 mL). The mixture was purged with O_2 for 5 minutes and flowed through the setup while stirring. To agitate the reaction, DIC was added through the septum via micro syringe at indicated time points and/or the light source was turned on/off. Upon DIC addition, the light source was OFF for 5 minutes to avoid DIC decomposition. IR spectra were recorded at 1 spectrum per minute intervals.

After reaction completion, 900 µL of the reaction mixture was added to a known amount of 1,3,5-trimethoxybenzene in CH_3CN (100 µL). After shaking, the mixture was analyzed by GC-MS and by FID-integration versus the internal standard, the yield was determined using a calibration curve.

Data processing

The obtained IR spectra were exported as single channels and the background spectrum was kept the same for all spectra, which was a spectrum containing only CD_3CN/D_2O in a 4/1 ratio. From the resulting absorbance spectra, the region between 1988 and 1670 cm^{-1} was taken and they were set on zero at 1900 cm^{-1}. Then, to correct for the increasing H_2O absorption, the spectra were corrected with a Gaussian centered at the water band at 1640 cm^{-1} using a Matlab script. The final spectra between 1850-1670 cm^{-1} were used for the PLS model. For the first datapoint of each set, the starting concentrations were also provided to the model. PLS was performed in Origin, with method used SVD with 15 maximum factors and cross validated. Resulting diagnostics and scores plots are shown below.

Training set

As training set for the PLS model, several combinations of acid **3**, ketone **4** and anhydride **5** in CD_3CN/D_2O (4/1) were measured with FT-IR and the spectra after processing described above were used to train the model (Table S7). This gave the resulting diagnostics for each component.

Table S7: Concentrations of components that were measured by FT-IR, used to train the PLS model.

Entry	Ketone **4** [mM]	Acid **3** [mM]	Anhydride **5** [mM]
1	0	0	5.115
2	0	1	4.064
3	0	2	2.872
4	0	3	1.788
5	0	4	0.846
6	0	5	0
7	1	0	4.076
8	1	1	2.969
9	1	2	2.061
10	1	3	1.033
11	1	4	0
12	2	0	3.236
13	2	1	2.066
14	2	2	1.243
15	2	3	0
16	0	2.5	2.611
17	1.5	1.5	2.117
18	2	0.5	2.918
19	1	0	4.161
20	5	0	0

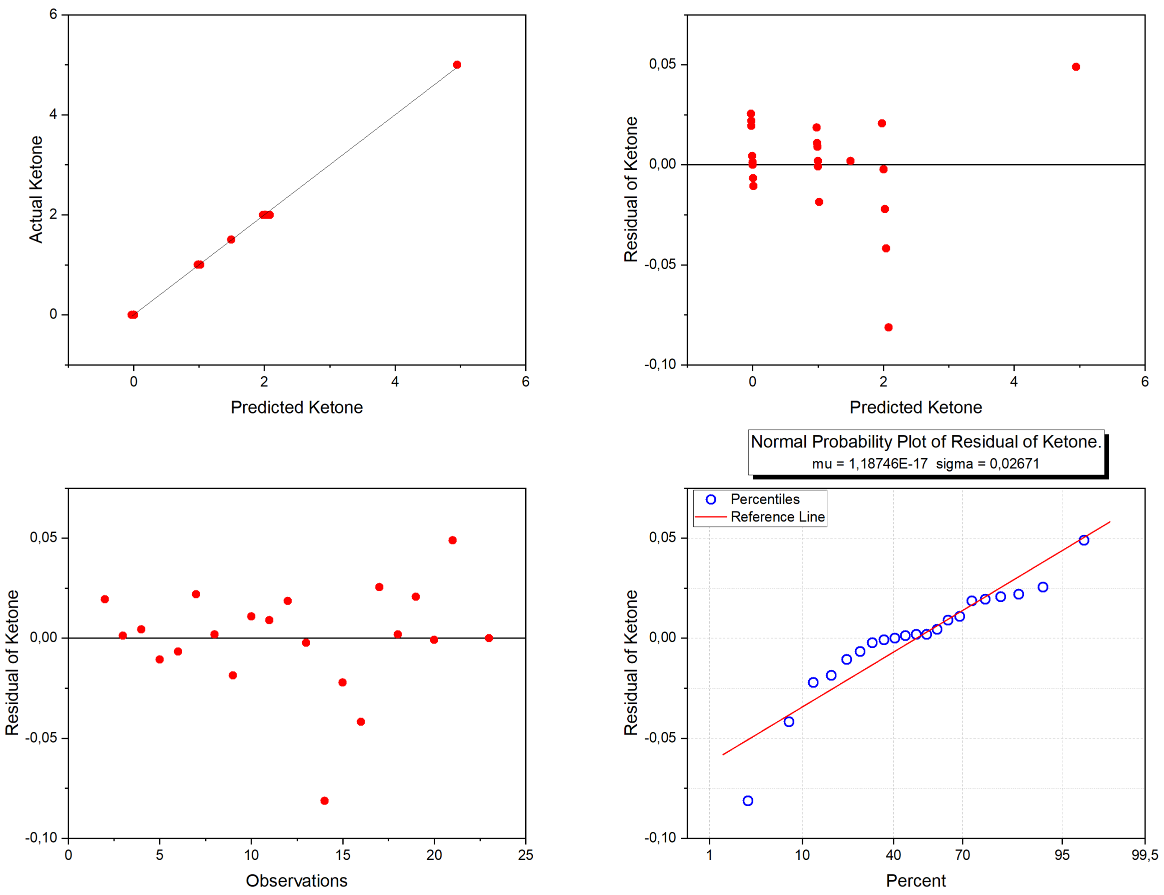

*Figure S31: Diagnostics for ketone **4** quantification of the PLS model.*

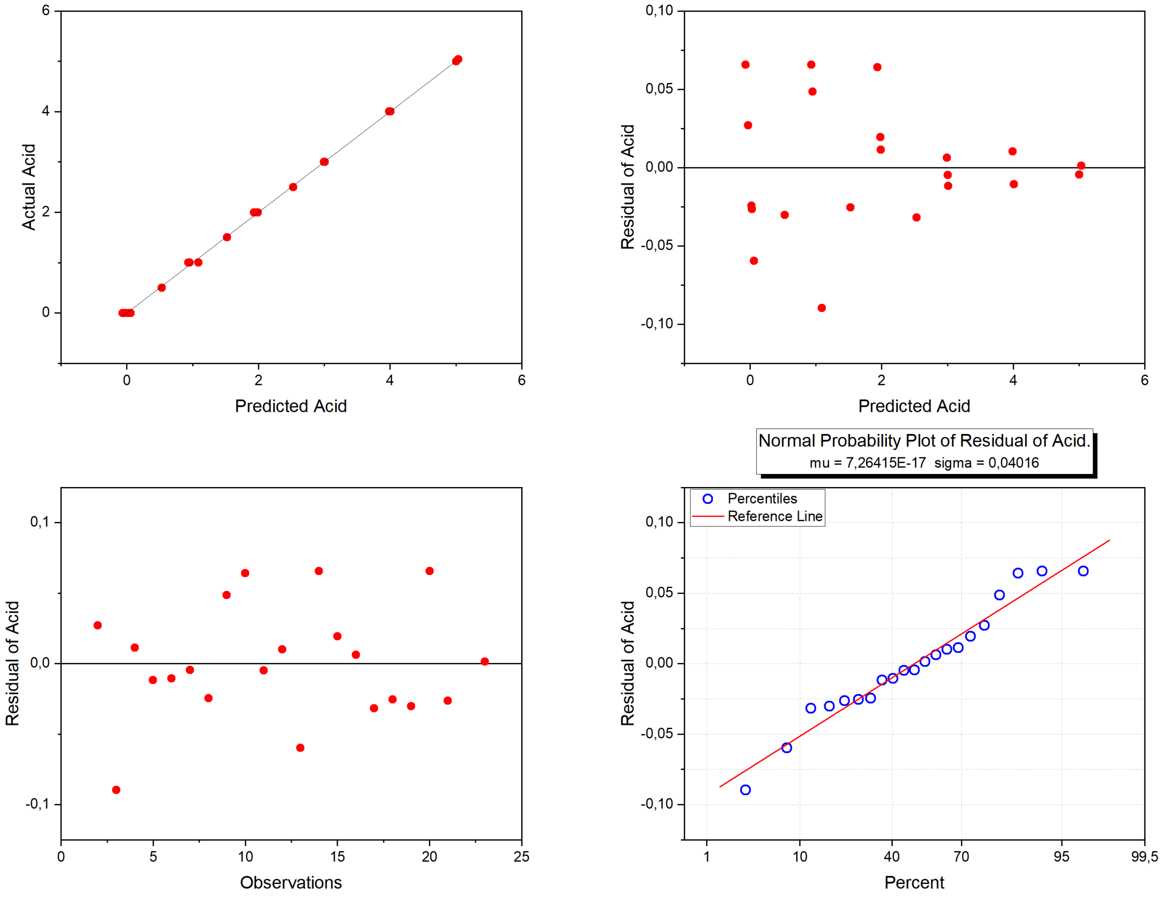

*Figure S32: Diagnostics for acid **3** quantification of the PLS model.*

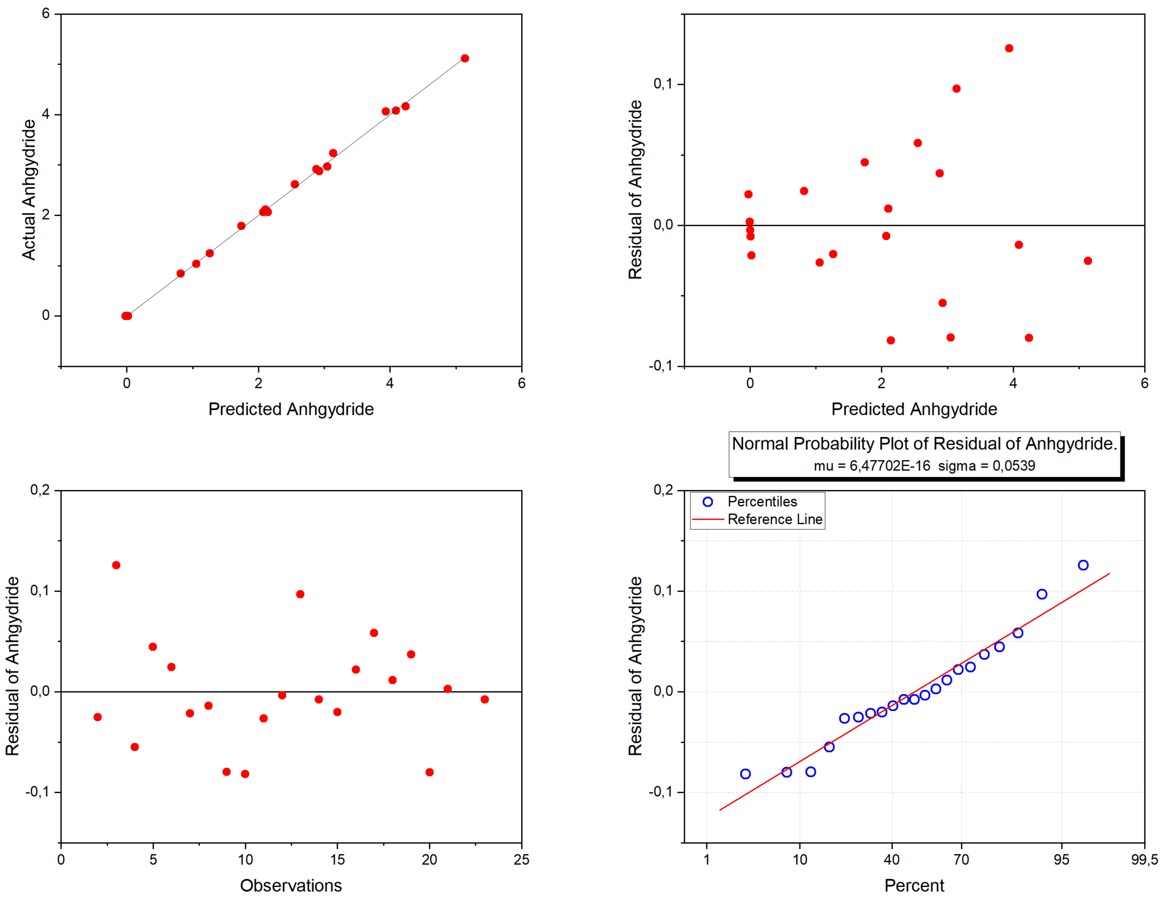

*Figure S33: Diagnostics for anhydride **5** quantification of the PLS model.*

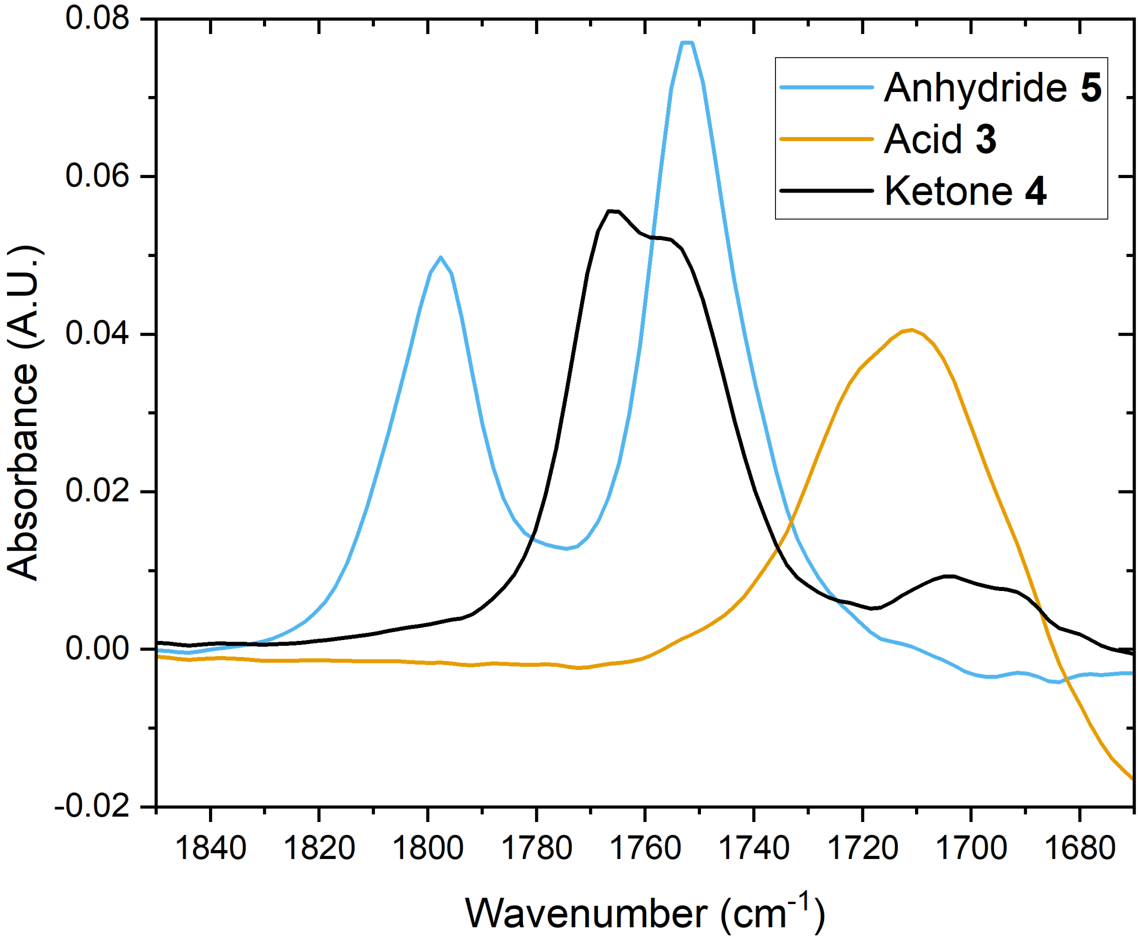

*Figure S34: FT-IR absorption spectra of 5 mM of pure compounds **3**, **4** and **5** in CD_3CN/D_2O 4/1 after processing as described above in the general procedure for FT-IR spectroscopy.*

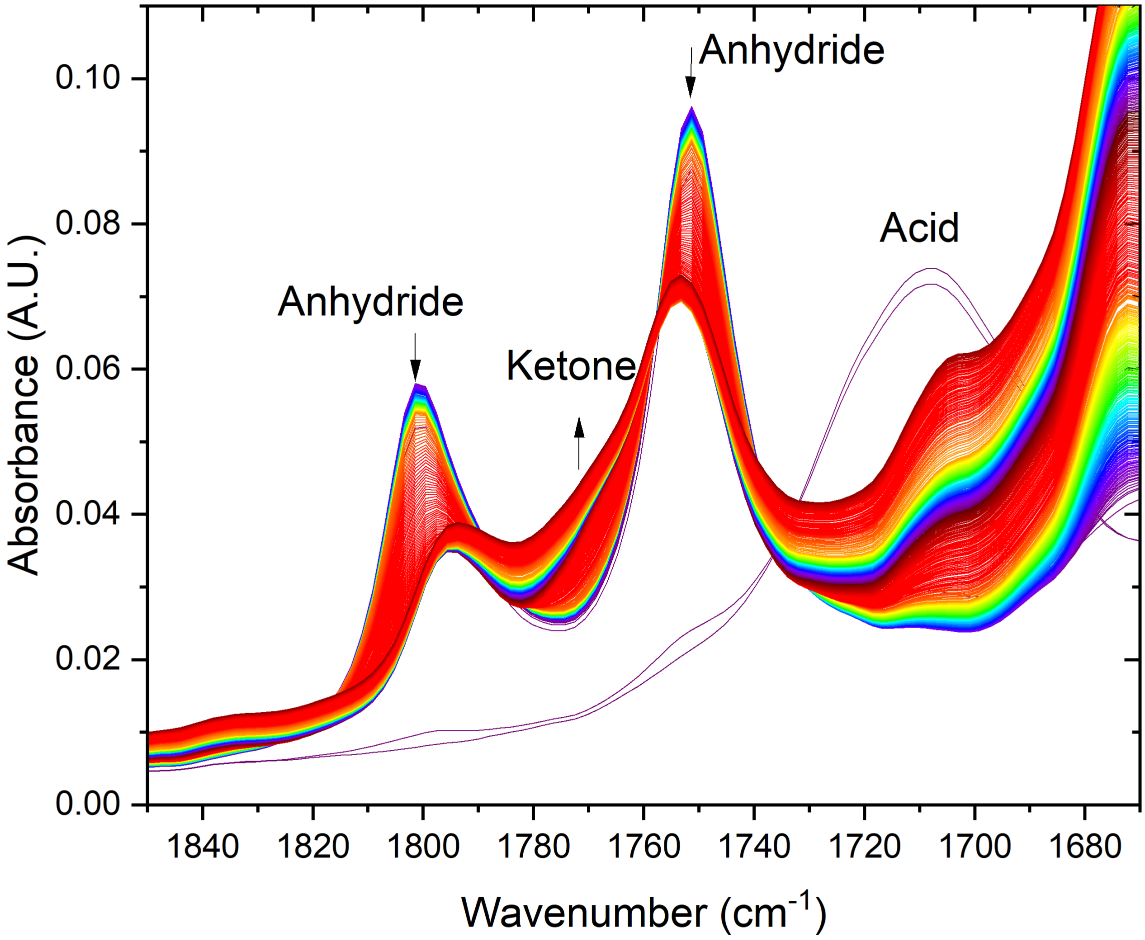

Figure S35: Typical FT-IR absorption spectra which were obtained without H_2O correction. Water absorption increases in time at the left edge of the spectrum (centered around 1640 cm^{-1}) which is still clearly visible. Spectra are recorded over time, starting with purple (acid only), going to red (etc). Initially, only acid is present, which is rapidly converted to anhydride upon DIC addition. Then, more slowly, the anhydride is converted to ketone (and acid).

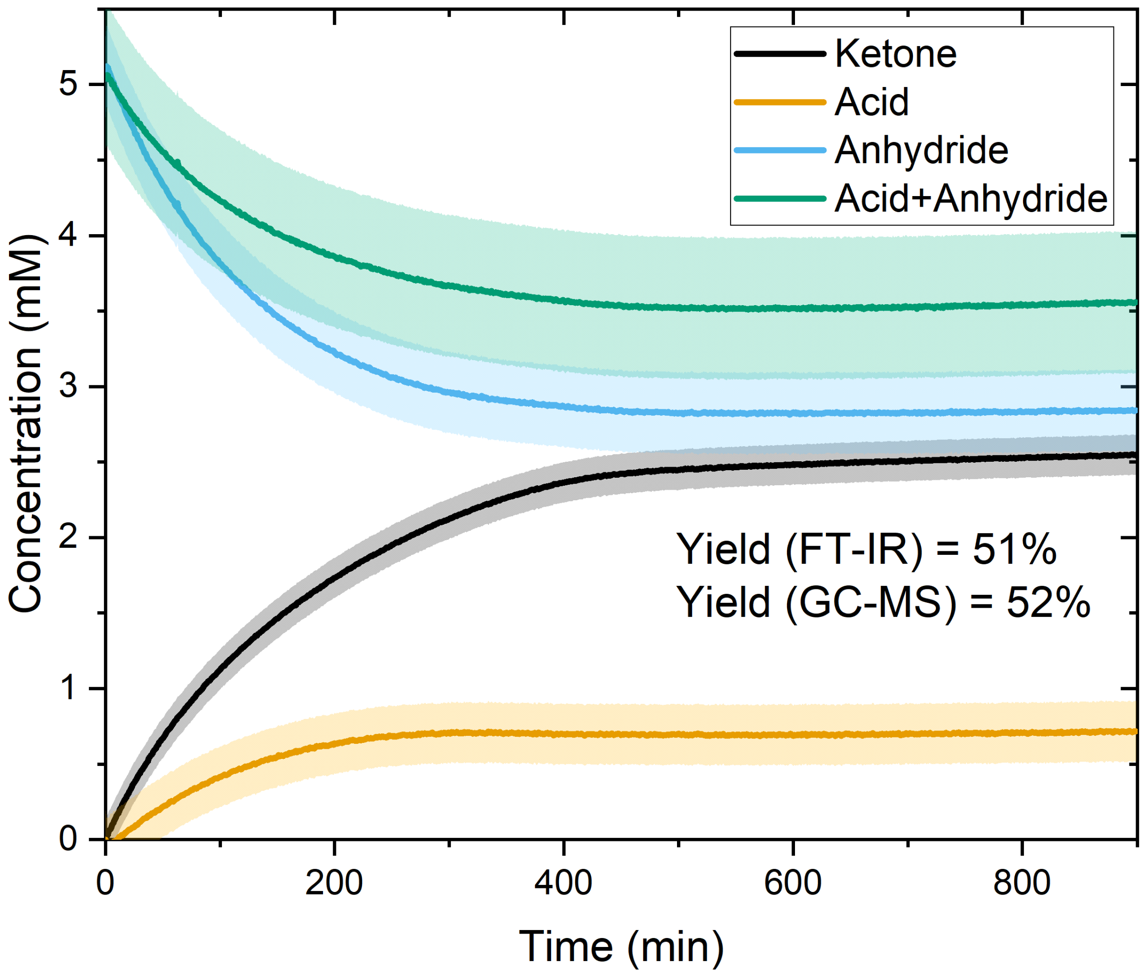

Figure S36: Reaction profile of decarboxylation of anhydride ***5*** *with 1 mol% Pt12LGua24 in the FT-IR setup using the general protocol for FT-IR as described above. Light source is continuously on during the experiment.*

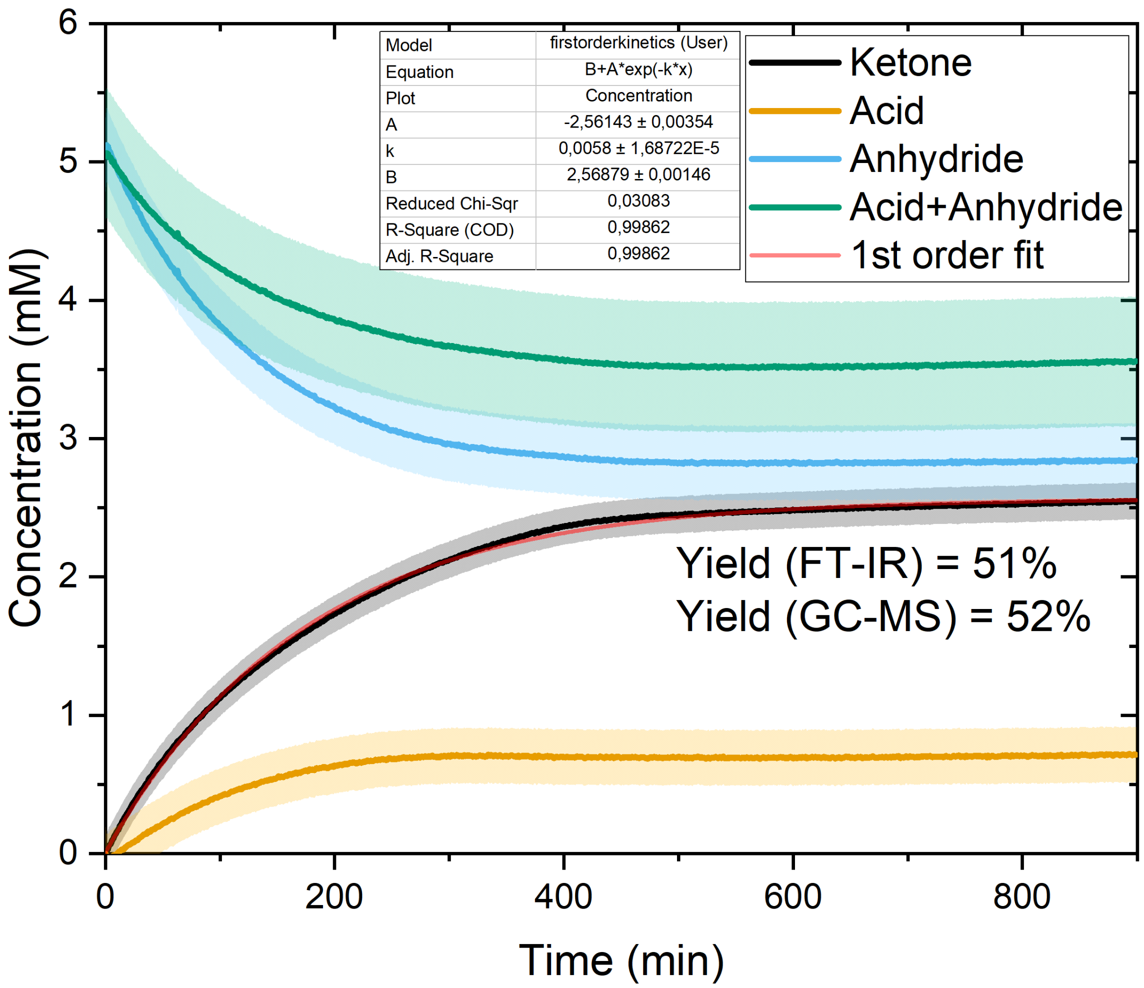

*Figure S37: Reaction profile (same as is shown in Figure S36) of decarboxylation of anhydride **5** with 1 mol% Pt12LGua24 in the FT-IR setup using the general protocol for FT-IR as described above. Light source is continuously on during the experiment. Plot contains first order first for production of ketone.*

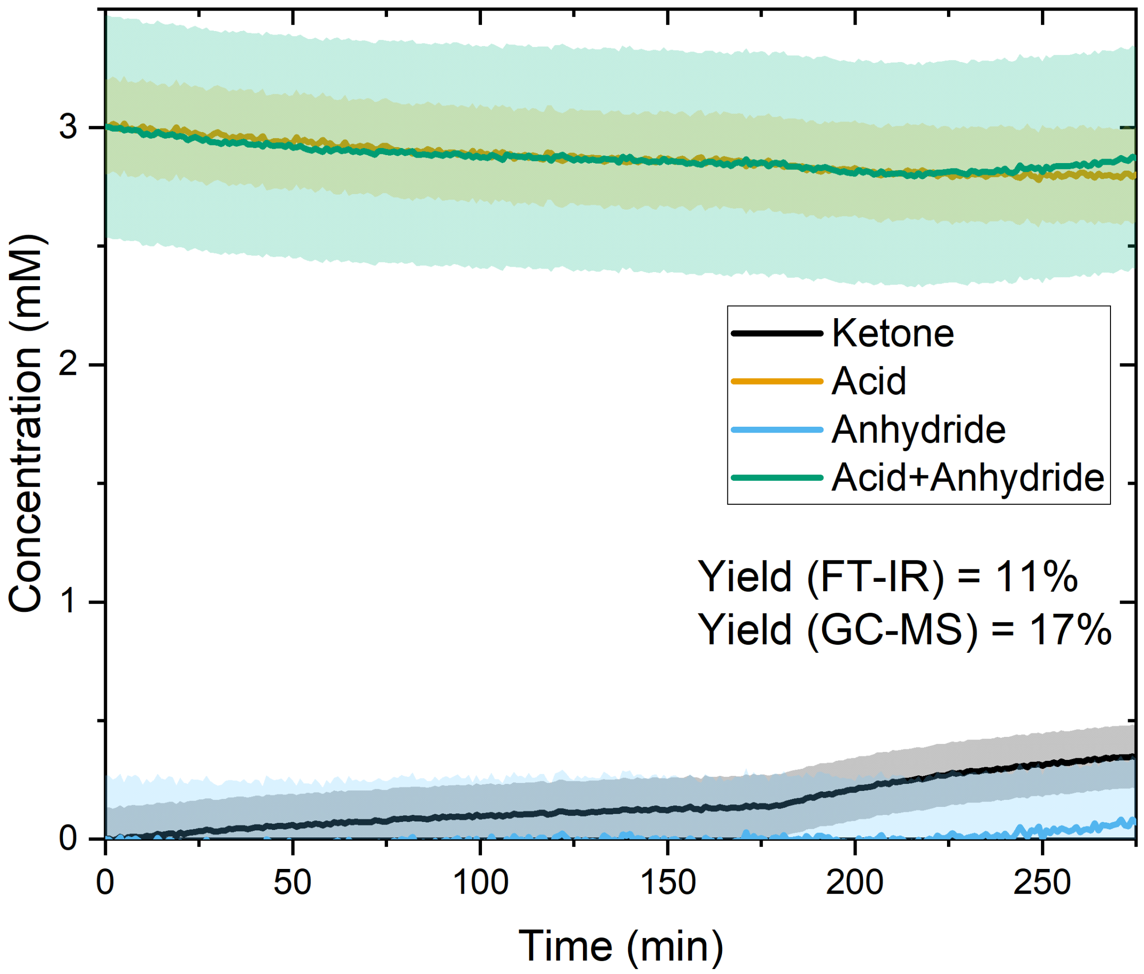

*Figure S38: Reaction profile of decarboxylation in OR mode with acid **3** (3 mM) and 1 mol% $Pt_{12}LGua_{24}$ in the FT-IR setup using otherwise the general protocol for FT-IR as described above without DIC. Light source is continuously on during the experiment.*

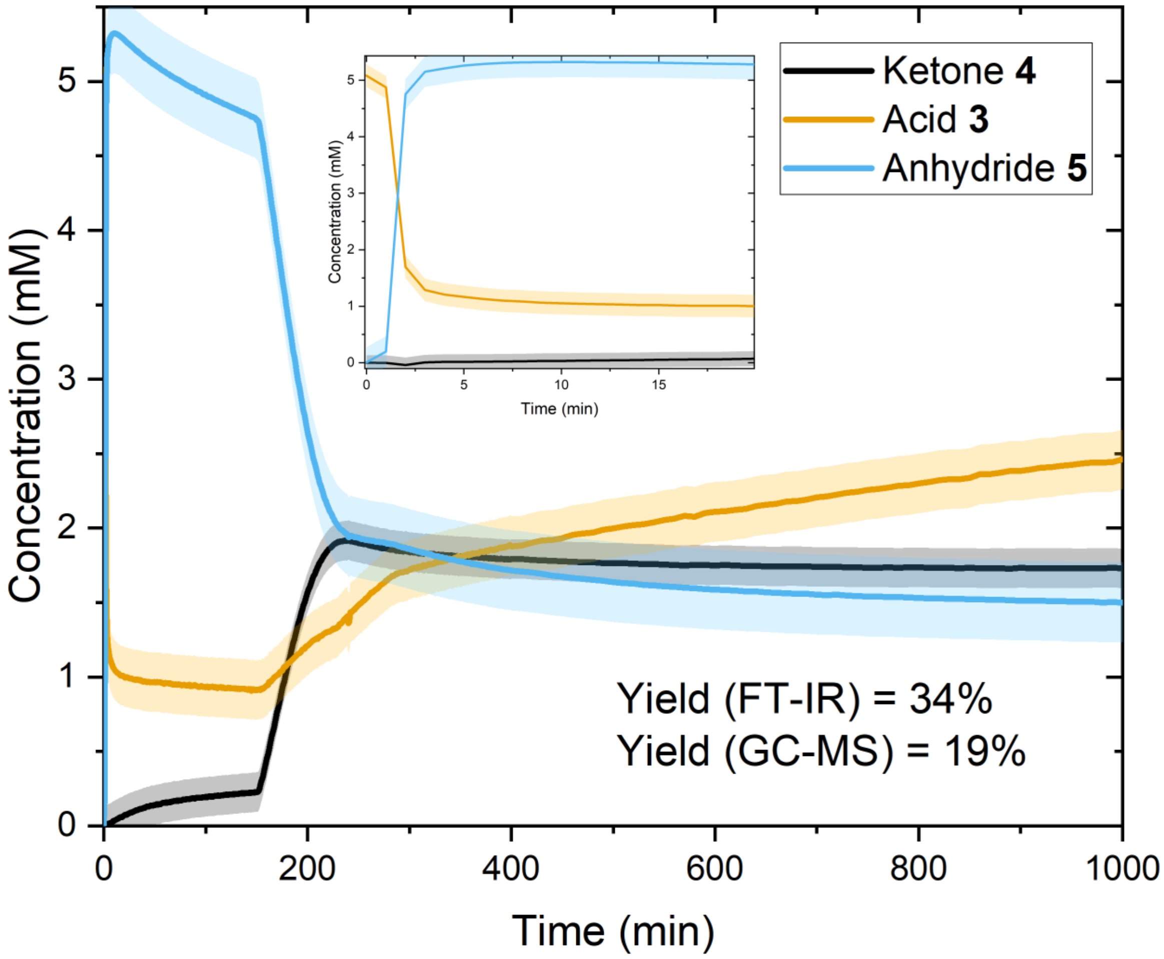

Figure S39: Reaction profile of decarboxylation in OR mode, switched to AND mode (at t = 150 min) with acid ***3*** *(5 mM) and 1 mol% $Pt_{12}LGua_{24}$ in the FT-IR setup using otherwise the general protocol for FT-IR as described above. DIC (2.0 eq.) were added at t = 0 min, shown in the zoom in of the first 20 minutes. The yield difference between IR and GC-MS is attributed to N-acylurea side-product formation.*

6. GC/MS calibration curves

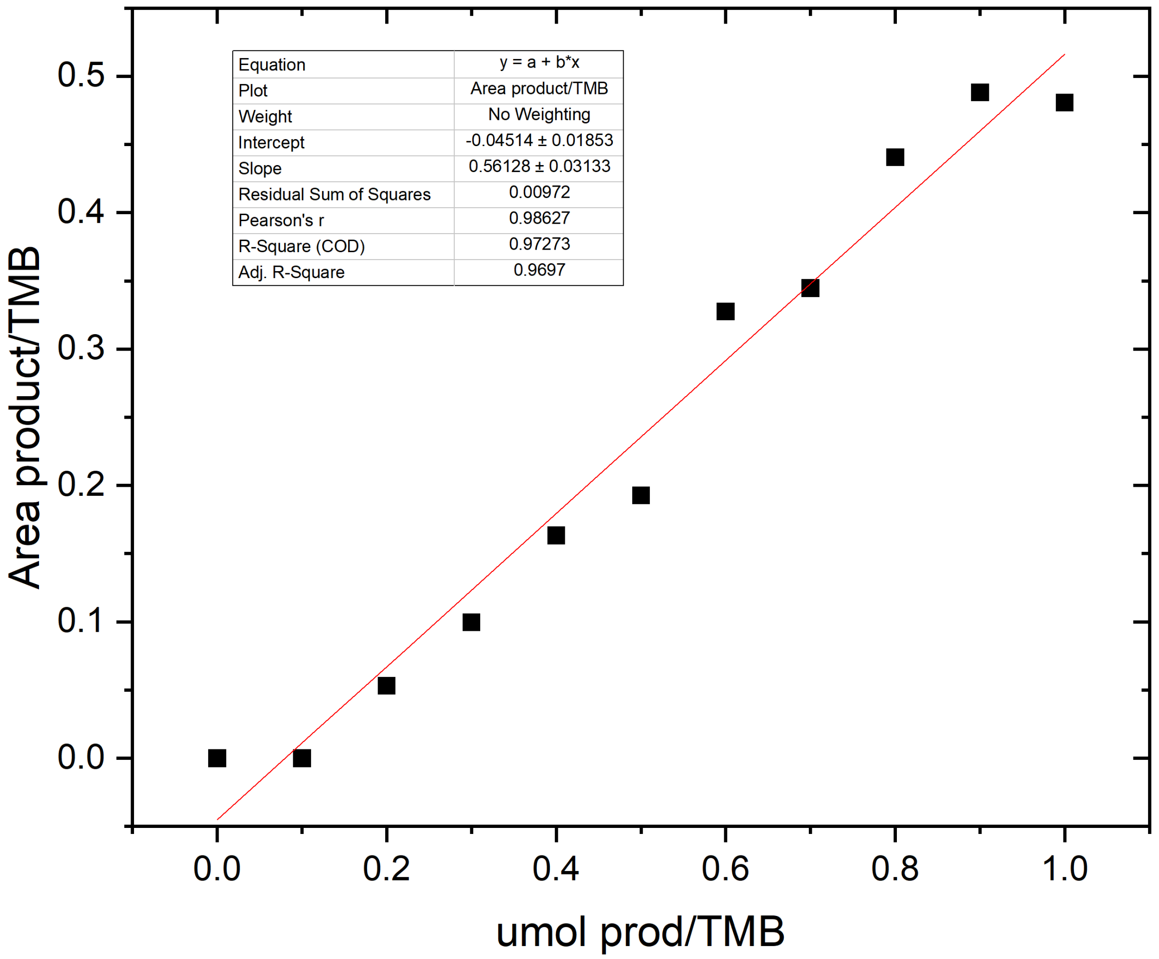

Figure S40: Calibration curve of 2-acetylbenzoic acid using 1,3,5-trimethoxybenzene as reference. Y-axis depicts the area ratio 2-acetylbenzoic acid/1,3,5-trimethoxybenzene (TMB) and the x-axis the µmol ratio 2-acetylbenzoic acid/1,3,5-trimethoxybenzene (TMB).

7. Experimental references

1 E. O. Bobylev, D. A. Poole III, B. de Bruin, J. N. H. Reek, *Chem. Sci.*, 2021, **12**, 7696–7705.

2 E. O. Bobylev, D. A. Poole, B. de Bruin, J. N. H. Reek, *Chem. Eur. J.*, 2021, **27**, 12667–12674.

3 R. Ham, B. Baumgartner, J. N. H. Reek, *Angew. Chem. Int. Ed.*, DOI:10.1002/anie.202508131.

4 M. Billamboz, F. Bailly, C. Lion, C. Calmels, M.-L. Andréola, M. Witvrouw, F. Christ, Z. Debyser, L. De Luca, A. Chimirri, P. Cotelle, *Eur. J. Med. Chem.*, 2011, **46**, 535–546.

5 R. Boda, G. D. Cuny, *Tetrahedron*, 2022, **129**, 133146.

6 Z. Ashbridge, J. N. H. Reek, *Angew. Chem. Int. Ed.*, DOI:10.1002/anie.202500214.

7 T. M. Masson, S. D. A. Zondag, J. H. A. Schuurmans, T. Noël, *React. Chem. Eng.*, 2024, **9**, 2218–2225.
